# Tunable and Degradable Dynamic Thermosets from Compatibilized Polyhydroxyalkanoate Blends

**DOI:** 10.1021/acssuschemeng.5c00943

**Published:** 2025-02-27

**Authors:** Chen Ling, Ryan W. Clarke, Gloria Rosetto, Shu Xu, Robin M. Cywar, Dong Hyun Kim, Levi J. Hamernik, Stefan J. Haugen, William E. Michener, Sean P. Woodworth, Torrey M. Lind, Kelsey J. Ramirez, Meltem Urgun-Demirtas, Davinia Salvachúa, Christopher W. Johnson, Nicholas A. Rorrer, Gregg T. Beckham

**Affiliations:** † Renewable Resources and Enabling Sciences Center, National Renewable Energy Laboratory, Golden, Colorado 80401, United States; ‡ Agile BioFoundry, Emeryville, California 94608, United States; § BOTTLE Consortium, Golden, Colorado 80401, United States; ∥ Applied Materials Division, Argonne National Laboratory, Lemont, Illinois 60439, United States; ⊥ Northwestern Argonne Institute of Science & Engineering, Evanston, Illinois 60208, United States

**Keywords:** microbial synthesis, metabolic engineering, biobased polymer, PHA, vitrimer, biodegradable polymer

## Abstract

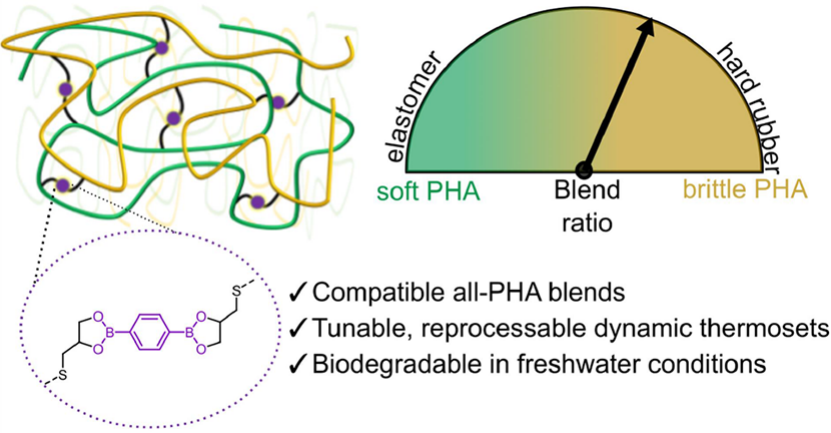

Polyhydroxyalkanoates (PHAs) are versatile, biobased polyesters that are often targeted for use as degradable thermoplastic replacements for polyolefins. Given the substantial chemical diversity of PHA, their potential as cross-linked polymers could also enable similar platforms for reversible, degradable thermosets. In this work, we genetically engineered *Pseudomonas putida* KT2440 to synthesize poly(3-hydroxybutyrate-*co*-3-hydroxyundecenoate) (PHBU), which contains both 3-hydroxybutyrate and unsaturated 3-hydroxyundecenoate components. To reduce the brittleness of this polymer, we physically blended PHBU with the soft copolymer poly(3-hydroxydecanonate-*co*-3-hydroxyundecenoate) in mass ratios of 1:3, 1:1, and 3:1. Upon observing varying degrees of immiscibility by scanning electron microscopy, we installed dynamic boronic ester cross-links via thiol–ene click chemistry, which resulted in compatibilized dynamic thermoset blends ranging in hard, medium, and soft rubber or elastomer thermomechanical profiles. These dynamic thermoset blends were subjected to controlled biological degradation experiments in freshwater conditions, achieving timely mass loss despite the cross-linked architectures. Overall, this work highlights a two-component platform for the production of degradable and reprocessable dynamic thermoset blends suitable for several classes of cross-linked polymer technologies from tailored, biological PHA copolymers.

## Abstract 2

Microbially produced polymers are cross-linked to form rubber-like materials. These biobased materials can be reprocessed and are biodegradable.

## Introduction

The global pollution crisis stemming from plastic waste accumulation in the natural environment is prompting demand for a circular material economy that relies on sustainable alternatives to conventional fossil carbon-based plastics.^1−4^ As a promising option, polyhydroxyalkanoates (PHAs) are a class of biodegradable polyesters that can be sourced from microbial synthesis using renewable feedstocks.^5−10^ PHA side chains are highly tunable via biological and chemical approaches, and this versatility has been leveraged to obtain thermoplastics with enhanced thermal stability, polyolefin-like properties, and catalytic depolymerization to monomers.^2,11−17^ Generally, short-chain-length (*scl*) PHAs consisting of pendent groups of 1–2 carbon atoms are rigid, medium-chain-length (*mcl*) PHAs with pendent 3–11 carbon atoms are often soft materials exhibiting elastomeric tensile profiles,^18,19^ and longer chain length PHAs (pendent groups >11 carbon atoms) are less common but generally softer.^7^

**Figure 1:**
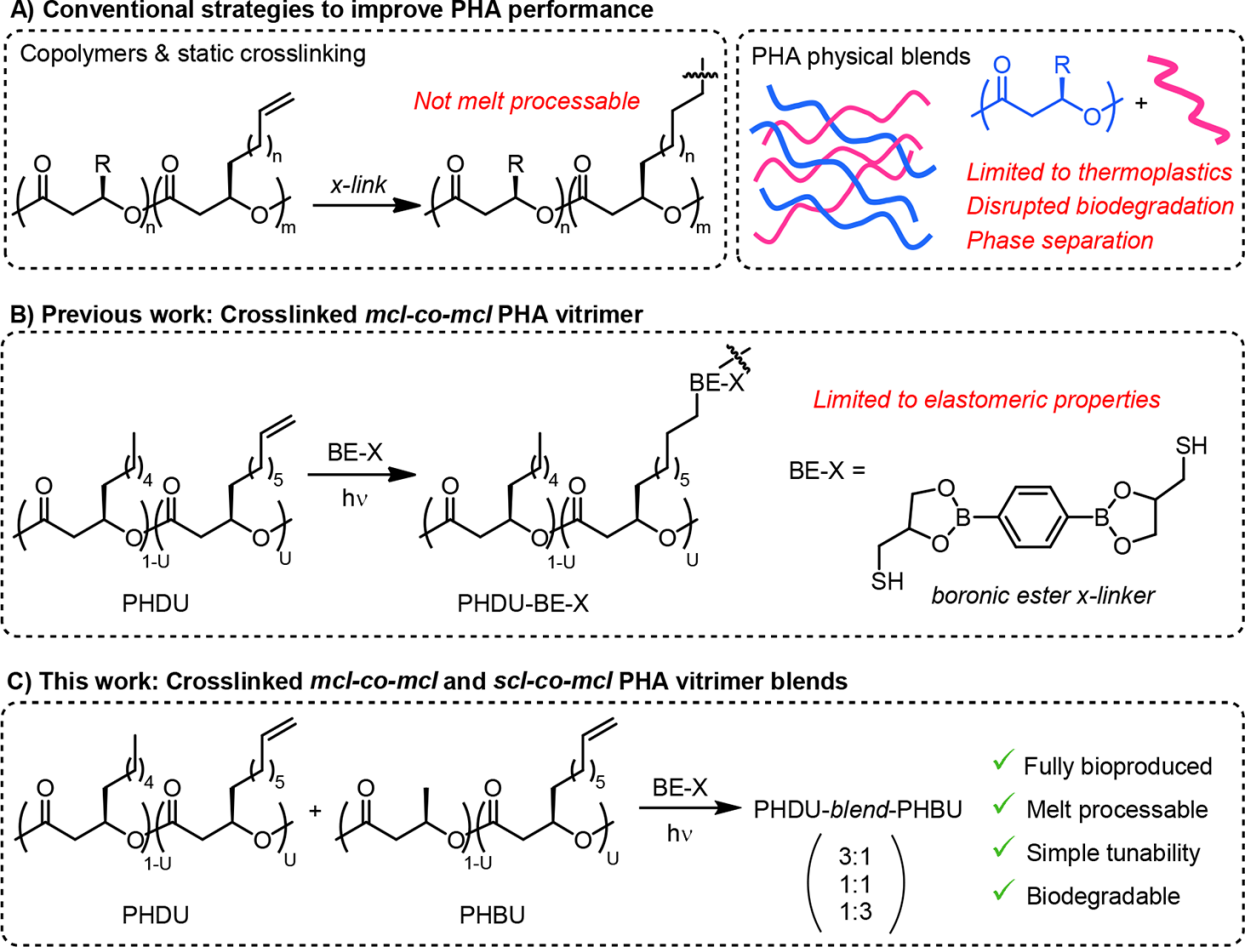
Overview of past efforts in PHA thermoset materials and advances in this work. (A, B) Previously reported strategies to enhance PHA thermal and mechanical performance, and (C) herein-reported strategy to obtain tunable, degradable thermoset materials. PHA, polyhydroxyalkanoates; PHDU, poly(3-hydroxydecanoate-*co*-3-hydroxyundecenoate); BE-X, boronic ester cross-linker 2,2-(1,4-phenylene)-bis[4-thioethyl-1,3,2-dioxaborolane]; and PHBU, poly(3-hydroxybutyrate-*co*-3-hydroxyundecenoate).

To date, advances in designer-PHA materials have been primarily directed toward thermoplastics. While thermoplastics encompass the majority of plastic production, thermosets (cross-linked polymers) comprise 18% of the annual polymer manufacture and often present a greater challenge for enabling end-of-life (EoL) technologies.^20−22^ PHAs are of interest in this space as biobased alternatives to synthetic elastomeric materials, and importantly, it has been shown that cross-links in PHA do not affect biodegradability.^23−25^ Microbially produced PHA copolymers with unsaturated pendent groups (denoted usPHAs) can be efficiently cross-linked with high energy irradiation,^23^ UV,^26^ thiol–ene click chemistry,^27−29^ or peroxides (Figure 1A).^30,31^ The resulting cross-linked products can serve as rubber materials,^32^ pressure-sensitive adhesives,^33^ and shape memory composites.^34^ However, the lack of melt processability, solubility, and thermomechanical reconfiguration upon cross-linking consequently precludes typical recycling approaches at EoL.^23,28,35^ Covalent adaptable networks (CANs) and vitrimers present an ideal alternative in this space, as thermally triggered dynamic bond exchange across cross-links allows for sufficient chain mobility for melt reprocessability at elevated temperatures while retaining thermoset performance at ambient temperatures. We recently reported an example involving an *mcl*-PHA, specifically poly(3-hydroxydecanonate-*co*-3-hydroxyundecenoate) (PHDU), chemically cross-linked with dynamic-covalent boronic ester linkages, resulting in the creation of elastomers that were both reprocessable and biodegradable in freshwater conditions (PHDU-BE-X, Figure 1B).^36^ The optimum properties for the copolymer were found to be at lower cross-linking densities (∼6%), which resulted in a soft elastomer. With additional design effort, dynamic cross-linked PHAs could serve as an entire materials platform, allowing a wider range of properties to be accessed.

Another route to improving the performance of PHAs is blending with other biopolymers, such as poly(ε-caprolactone), polysaccharides, natural rubber,^37,38^ and other PHAs (Figure 1A).^39^ A common complication is compatibility of the blends, which influences material performance, in turn requiring compatibilizers to facilitate miscibility and prevent phase separation over time. Nevertheless, melt mixing of poly(3-hydroxybutyrate-*co*-3-valerate) (PHBV) and poly(3-hydroxybutyrate-*co*-3-hydroxyhexanoate) at different ratios resulted in homogeneous blends, although the properties were largely dominated by PHBV and remained brittle, despite the addition of a more ductile PHA.^40^ A similar example between poly-3-hydroxybutyrate (P3HB) and *mcl*-PHA blends showed that improvements in material properties were only observed when nanocellulose was added to the matrix.^41^ More generally, dynamic cross-linking has been demonstrated as a means to upcycle a broad range of immiscible plastic waste mixtures to dynamic thermoset blends with full compatibility.^42^ Considering these examples, here, we posited that PHAs with different thermomechanical profiles could also be blended and compatibilized via dynamic cross-links to yield favorable performance and EoL biodegradability.

In this work, we replaced the native *mcl*-specific PHA synthases in *Pseudomonas putida* KT2440 (hereafter *P. putida*) with a heterologous synthase,^43,44^ allowing the engineered strain to produce *scl*-*co*-*mcl* PHA copolymers, thus expanding the range of mechanical properties compared to *mcl* PHAs alone. We subsequently demonstrated that through the physical blending of *scl*-*co-mcl* and *mcl*-*co*-*mcl* usPHAs, along with the promotion of compatibilization via dynamic boronic ester cross-linking at the pendent alkenes, it is possible to achieve a high degree of control over the modulus and extensibility of the resulting network polymers. EoL options were investigated by subjecting each of the substrates to biodegradability experiments in freshwater conditions, highlighting retained biodegradation properties despite the incorporation of cross-links. Thus, this work affords a straightforward platform to obtain several classes of sustainable thermoset materials by variation of the blend composition.

## Results

### Introduction of PhaC_61-3_-S325T/Q481K to *P. putida* for *scl*-*co*-*mcl* PHA Production from Structurally Related Fatty Acids

**Figure 2:**
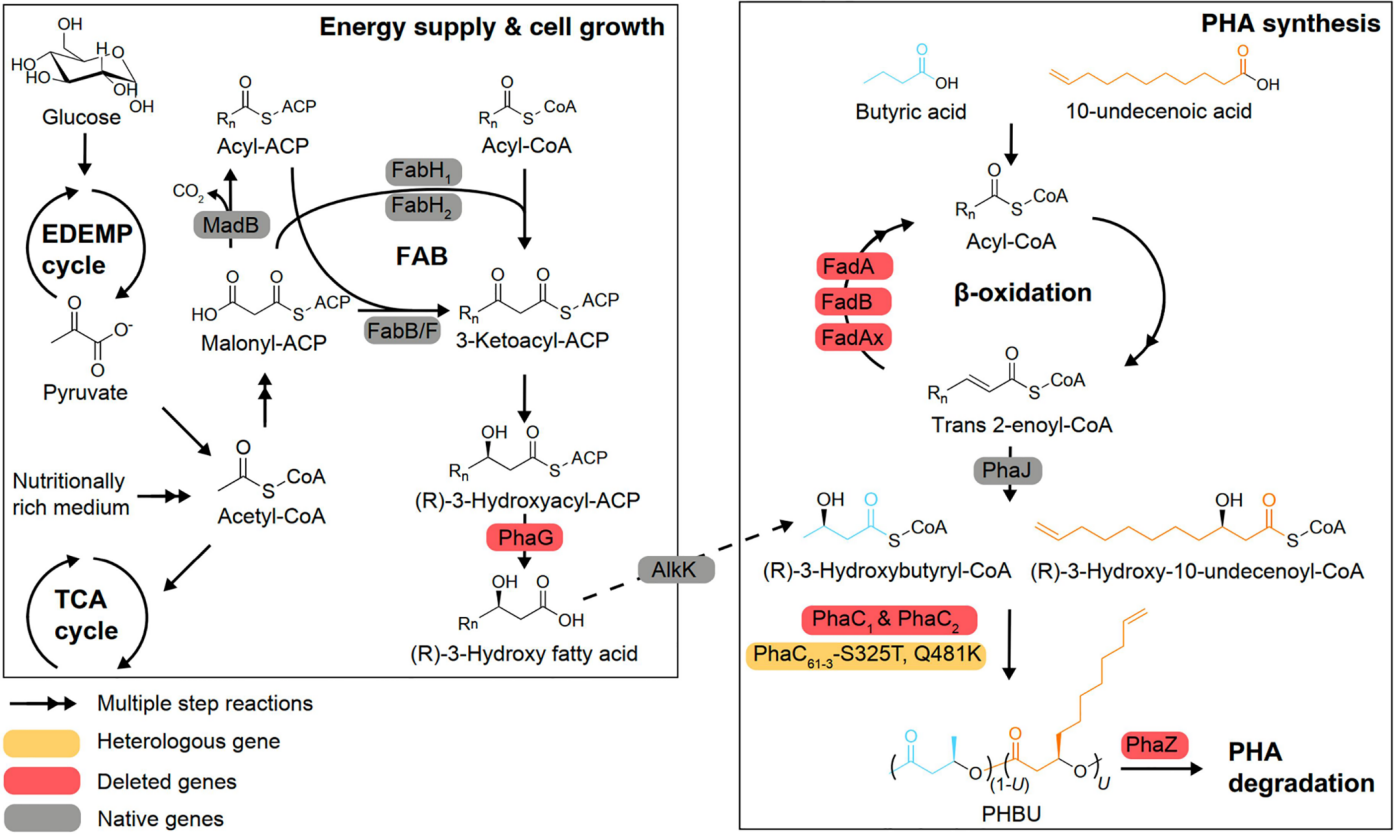
Pathway of cellular metabolism (left) and PHBU production (right) in *P. putida* LC059. Several genes in the β-oxidation pathway were deleted to prevent degradation of *trans* 2-enoyl-CoA. The gene *phaG* was knocked out to prevent the flux from FAB to PHA synthesis. Glucose was supplemented to supply energy and biomass formation. The native type II PHA synthases, PhaC_1_ and PhaC_2_, were replaced with PhaC_61-3_-S325T/Q481K to accept a broader range of 3-hydroxyacyl-CoA as substrates to produce the desired PHA copolymers. The gene encoding native PHA depolymerase, *phaZ*, was knocked out to prevent the degradation of PHBU.

To produce *scl*-*co*-*mcl* PHAs in *P. putida*, we pursued two primary goals. First, we aimed to replace the native *mcl*-specific PHA synthases with a more versatile variant capable of polymerizing both the *mcl* and *scl* precursors. Second, we sought to attenuate fatty acid degradation by selectively deleting genes within the β-oxidation pathway, thus ensuring the preservation of fatty acid substrates that were ultimately provided to the engineered strain for tailored PHA production. Consequently, the supplied fatty acid substrates—sodium butyrate and 10-undecenoic acid—were directed toward forming *mcl* and *scl* blocks in the PHA product, and an alternative carbon source (here, a nutritionally rich medium or glucose) was required for energy and cell growth. The strain engineering approach of deleting gene *phaG* to separate product synthesis from carbon and energy supply is illustrated in Figure 2.

As a starting point, we began with the engineered strain LC039 (Table 1) that is engineered to produce PHDU.^36^ Strain LC039 harbors deletion of several genes associated with fatty acid degradation (*fadAB*, *fabAx, fabBx*1*x*2, PP_2134–2135, PP_2218, and PP_2047–2051) to prevent degradation of exogenously provided fatty acid substrates. Additionally, the gene encoding PhaG was deleted to block the endogenous generation of 3-hydroxyacyl-CoAs via fatty acid biosynthesis (FAB), which could otherwise be incorporated into PHAs. Subsequently, we deleted the native *phaC*_*1*_*-phaZ-phaC*_*2*_ operon encoding two *mcl*-specific synthases, PhaC_1_ and PhaC_2_, and the PHA depolymerase PhaZ.^45^ Subsquently, we replaced this operon with a gene encoding a PHA synthase PhaC_61-3_-S325T/Q481K that is capable of polymerizing both *mcl* and *scl* units.^43,44^ The resultant strain LC059 (Table 1 and Figure 2) was utilized in the subsequent 500 mL and 2.8 L shake flask and 3 L bioreactor experiments to produce PHBU. Sodium butyrate and 10-undecenoic acid served as the substrates for PHBU synthesis, while a rich medium LB or mineral medium supplemented with glucose was employed to facilitate cell growth and energy consumption (Figure 2).

**Table 1:** Genotypes of the Primary Strains Used in This Study

strain	genotype	reference or source
LC039	*P. putida* KT2440 Δ*fadAB* ΔPP_2134–2135 Δ*fadBx*1 Δ*fadAx* Δ*fadBx*2 ΔPP_2218 Δ*phaG* ΔPP_2047-PP_2051	(36)
LC059	LC039 Δ*phaC*_*1*_*phaZphaC*_*2*_::P*_tac_*:*phaC*_61–3_-S325T/Q481K-codon optimized	this study

### Shake Flask and Bioreactor Cultivation for PHBU Production

To verify the functionality of PhaC_61-3_-S325T/Q481K in *P. putida* LC059, we first conducted shake flask experiments in Miller’s lysogeny broth (LB), a nutritionally rich medium, supplemented with 10-undecenoic acid and sodium butyrate as substrates to test the production of PHBU. Following the shake flask cultivations, the cells were subjected to centrifugation, washing, and lyophilization. The resulting dry cell mass was subjected to acidic methanolysis, converting the PHA product to 3-hydroxy acid methyl esters (HAMEs) for analysis. The resulting HAMEs were quantified using gas chromatography–mass spectrometry (GC/MS) to ascertain the monomer composition of the PHA copolymers.^45^

**Figure 3:**
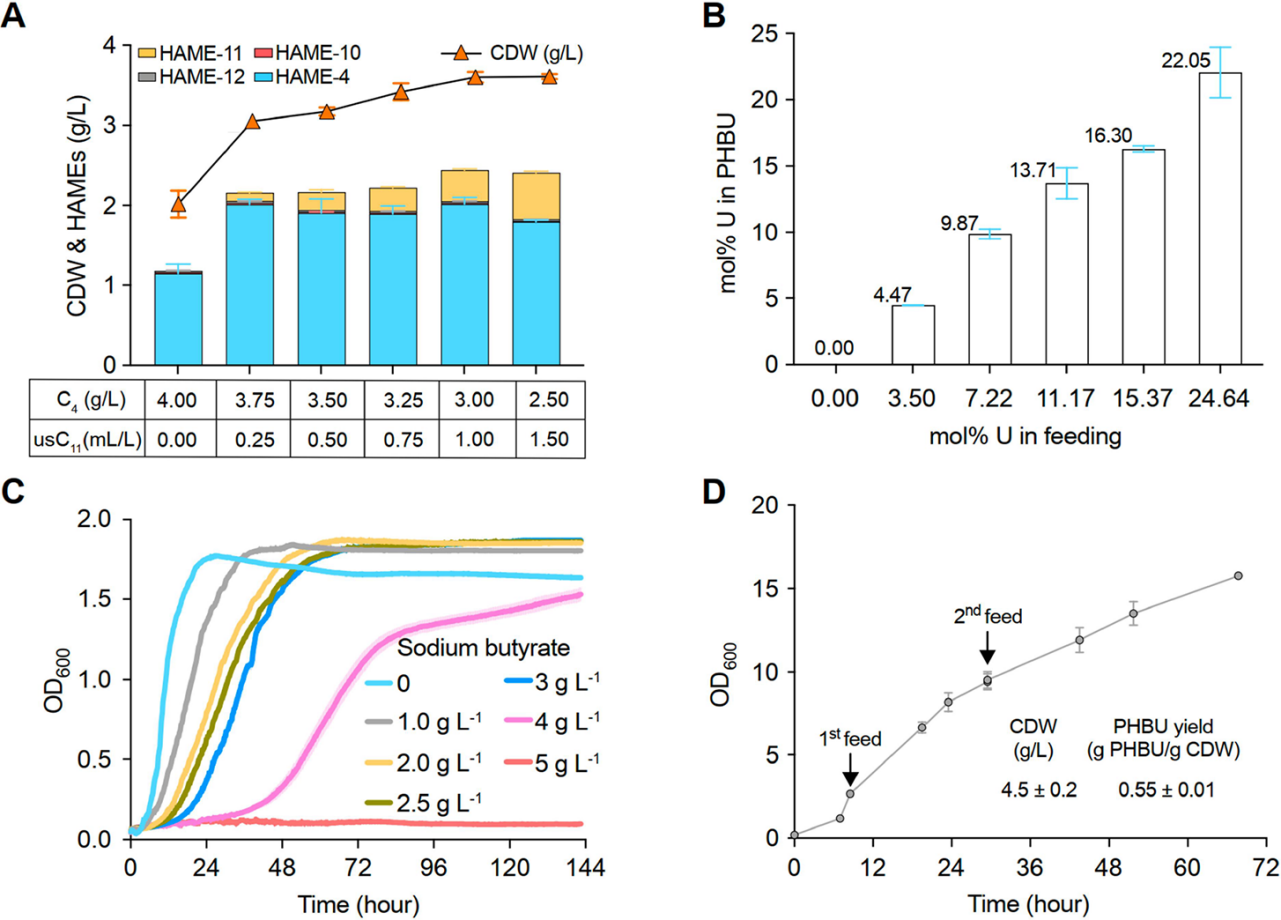
PHBU production in shake flask and bioreactor cultivations. (A) CDW and HAME profiles in shake flask cultivation samples. HAME profiles were analyzed by using GC/MS. C_4_ represents sodium butyrate, and usC_11_ represents 10-undecenoic acid. The concentrations of usC_11_ were calculated based on the density value of 0.912 g mL^–1^ at 25 °C, considering volume concentrations of 0, 0.25, 0.5, 0.75, 1.00, and 1.50 mL L^–1^. Error bars represent the standard deviation calculated from biological triplicates. The GC/MS plots for the analysis of HAME profiles have been incorporated in Figure S1C,D. (B) Quantification of the mol % U in PHBU using ^1^H NMR spectroscopy (Figure S4). The mol % values of feeding 10-undecenoic acid were corrected using the density value 0.912 g mL^–1^ at 25 °C. Error bars represent the standard deviation calculated from biological triplicates. (C) Growth curves of LC059 on M9 medium supplemented with 50 mM glucose, and in the absence or the presence of different sodium butyrate concentrations (1, 2.5, 5 g L^–1^). Error bars represent the standard deviation calculated from biological triplicates. (D) Optical density (OD_600_) and production of PHBU in 3 L bioreactors in the dissolved oxygen (DO)-stat fed-batch mode using modified M9 medium and glucose. We note that optical density does not necessarily correlate with growth due to PHA accumulation.^46^ The results are the average of two biological replicates, and the error bars represent the absolute difference between replicates. Black arrows indicate the time at which sodium butyrate (2 g L^–1^) and 10-undecenoic acid (0.16 g L^–1^) were added in the bioreactors.

In general, 3HB (HAME-4 in the ester form) constituted the main component of PHA accumulated by the strain LC059 across all six conditions, varying the concentrations of sodium butyrate and 10-undecenoic acid (Figure 3A). The fraction of 3-hydroxyundecenoate (HAME-11 in the ester form) increased as the ratio of 10-undecenoic acid to sodium butyrate increased, as expected (Figure 3A). Surprisingly, a small fraction of HAME-10 and HAME-12 was detected, each with a mass ratio of less than 2% (Figure 3A), which may be associated with unidentified fatty acid degradation and/or synthesis mechanisms.^36^ To determine the molar ratio of 3-hydroxyundecenoate (% U) in PHBU, the material was first extracted and purified to form a PHBU film, and the % U in these PHBU films was subsequently determined by ^1^H NMR spectroscopy (Figure S4), revealing a linear correlation with the mol % undecanoic acid fed as a substrate (Figure 3B). Notably, at the highest sodium butyrate concentration tested (4 g L^–1^), the total sodium butyrate utilization and cell dry weight (CDW) were the lowest (Figures 3A and S1A). We thus hypothesized that sodium butyrate might become toxic at approximately 4 g L^–1^. To examine this, we evaluated the cell growth of LC059 on a modified mineral (M9) medium supplemented with different concentrations of sodium butyrate. Our results showed that higher concentrations of sodium butyrate generally impair cell growth, with a notable decline observed between 3 and 4 g L^–1^, and no growth at 5 g L^–1^ (Figure 3C). These findings suggest that 4 g L^–1^ may represent a critical toxicity threshold (Figure 3C). Therefore, to avoid any growth inhibition by sodium butyrate, we chose to maintain its concentration at or below 2 g L^–1^ in subsequent experiments. In addition, incomplete consumption of 10-undecenoic acid and a lower utilization of sodium butyrate were observed when 1.37 g L^–1^ 10-undecenoic acid was supplemented (Figure S1A), suggesting that excess 10-undecenoic acid may also negatively affect sodium butyrate utilization. The conversion extent of 10-undecenoic acid to PHBU reached 40–45%, while sodium butyrate achieved nearly 100% (Figure S1B). The absence of C_9_ or other odd-numbered units suggests no β-oxidation degradation, with the low conversion efficiency of 10-undecenoic acid likely being attributed to its limited solubility or alternative oxidation pathways in *P. putida*.

We previously observed increased brittleness in PHDU materials when cross-linking exceeded 6 mol %,^36^ prompting us to target approximately 5 mol % unsaturation in our subsequent efforts producing PHBU. To assess the scalability of our system, we conducted PHBU production in 2.8 L shake flasks containing 500 mL of rich medium. The CDW remained consistent with our previous results on a smaller scale with 500 mL flasks containing 100 mL of medium, each producing ∼2–3 g L^–1^ (Figures S2 and 3A). We then combined the dry cells from six 2.8 L flasks (3 L cell culture in total) and extracted the materials, obtaining 6.40 g of purified *scl*-*co*-*mcl* PHAs (Figure S2).

To generate larger quantities of material for subsequent characterization, we conducted 3 L bioreactor cultivations using a DO-stat fed-batch strategy with the minimal M9 medium instead of a rich medium (see the Supporting Information). This feeding strategy allows the continuous and automatic addition of glucose—the sole carbon source—in the bioreactor while avoiding its accumulation and the generation of byproducts.^47^ Sodium butyrate and 10-undecenoic acid were manually added to the bioreactors at two different time points during the cultivations, at concentrations of 2 and 0.16 g L^–1^, respectively. Based on DO and agitation profiles (Figure S3), it is evident that the glucose uptake rate decreased after each pulse of butyrate and 10-undecenoic acid. This reflects a deceleration of metabolism due to the toxicity effect of these compounds and/or because of the accumulation of PHBU. We anticipate that future bioprocess optimization, involving pulsing substrates at lower concentrations over a longer duration, could reduce substrate toxicity and thereby increase the PHBU content (currently 55% CDW). 6.32 g of PHBU material extracted from the two bioreactors was combined with 6.40 g of materials extracted from the 2.8 L shake flasks, resulting in a pooled material with a mol % U of 5%, designated PHBU-5 (Figure S5).

### Characterization of PHBU-5 and PHBU-*blend*-PHDU Dynamic Thermoset Blends

**Figure 4:**
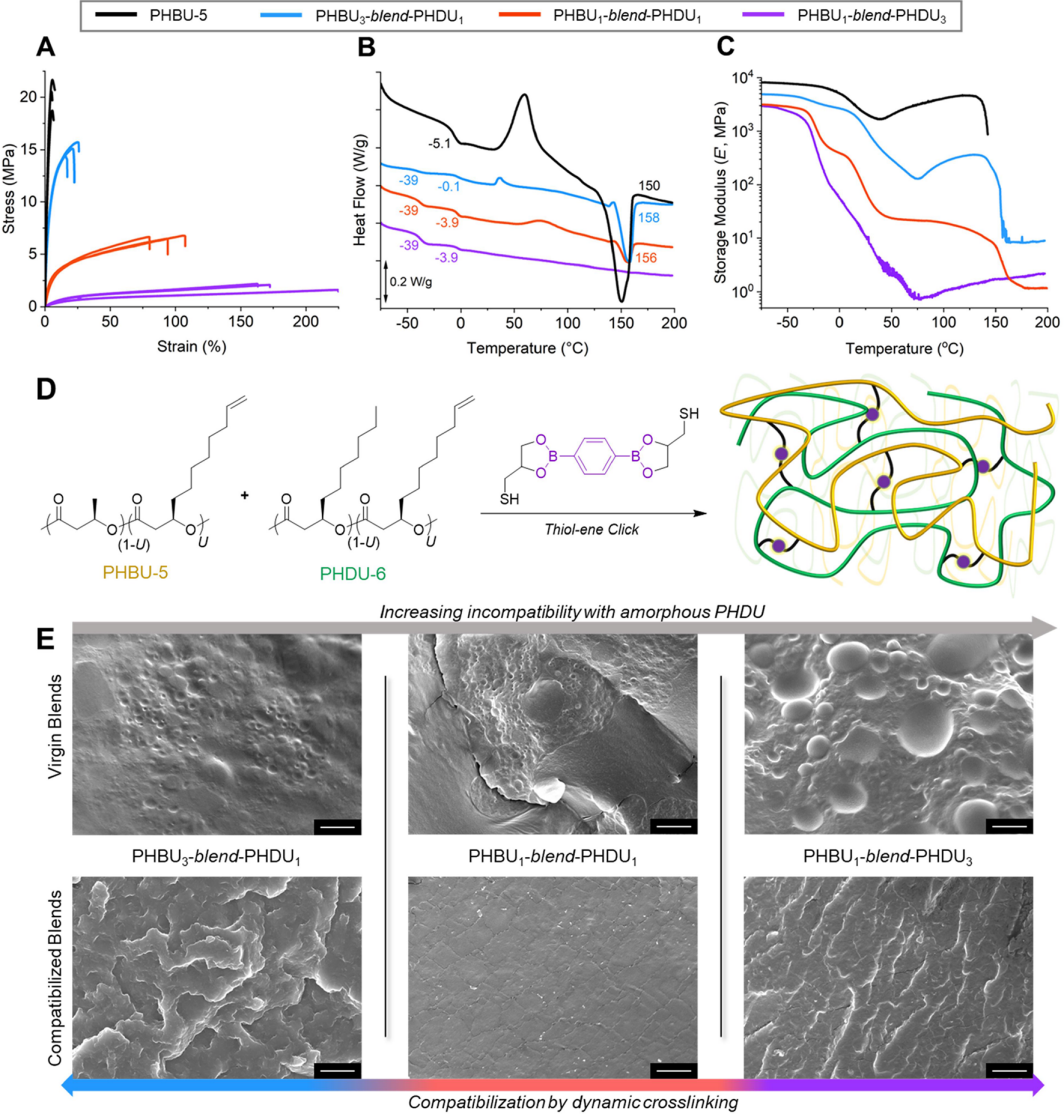
Preparation, compatibility, and mechanical analysis of hard-PHBU-5 and soft-PHDU-6 dynamic thermoset blends. (A) Representative tensile stress/strain tests (5 mm min^–1^, ∼23 °C) for PHBU-5 (black) and dynamically cross-linked PHBU-*blend*-PHDU at composition ratios by mass of 3:1 (blue), 1:1 (orange), and 1:3 (purple) (see Figure S9 for complete data and Table S5 for values). (B) Normalized DSC heat flow (10 °C min^–1^, exoup, first heating cycle) traces (see Figure S10 for individual plots and Table S6 for values). (C) DMA temperature-ramp frequency sweep thermograms (−75 to 200 °C, 3 °C min^–1^, 30 μm, 1 Hz) (see Figure S11 for individual thermographs and Table S7 for values), and (D) general scheme for the simultaneous blending and blend compatibilization by dynamic cross-linking of *scl*-*co*-*mcl* and *mcl*-*co*-*mcl* usPHAs to produce homogenized copolymer networks. (E) SEM cross-sectional imaging of virgin (top) and dynamic cross-link compatibilized (bottom) PHBU_3_-*blend*-PHDU_1_, PHBU_1_-*blend*-PHDU_1_, and PHBU_1_-*blend*-PHDU_3_ usPHA blends (scale bar = 15 μm) (see Figures S15–S17 for additional images and Figures S18–S20 for droplet diameters).

Recovered *scl*-*co*-*mcl* PHBU-5 was analyzed by ^1^H and ^13^C NMR and FTIR spectroscopies (Figures S5–S7), revealing that the polymer was of high purity as evidenced by the absence of the C_4_ and unsaturated C_11_ substrates. Additionally, gel permeation chromatography in CHCl_3_ returned a high molar mass product (*M*_n_ = 1.24 × 10^5^ kg mol^–1^, *Đ* = 2.2) (Figure S8). When subjected to tensile testing, PHBU-5 yielded similar qualities to that of the structurally similar homopolymer P3HB, namely, a brittle response with low elongations at break (ε_B_ = 2–5%).^36^ Otherwise, PHBU-5 exhibited a modest tensile strength (σ_B_ = ∼20 MPa) and Young’s modulus (*E* = 833 MPa) by tensile testing (Figures 4A and S9A, Table S5), along with high crystallinity reflected in the enthalpy of fusion (Δ*H*_f_) of 60 J g^–1^ at the melting temperature (*T*_m_) of 150 °C (Figure 4B). Crystallinity is limited by slow crystallization kinetics as indicated by the cold-crystallization peak (*T*_c_ ∼ 50 °C, Figure S10A), which could be overcome by thermal annealing. In addition, temperature-ramp frequency sweep thermograms by dynamic mechanical analysis (DMA) of PHBU agreed with thermal transitions measured by DSC, reflecting film failure approaching the *T*_m_ (∼145 °C), while also exhibiting storage modulus (*E*’) values of 8.1 GPa at −75 °C (*E*′_max_) and 2.3 GPa at ∼23 °C (*E*′_RT_) (−75 to 150 °C, 3 °C min^–1^, 30 μm, 1 Hz, Figures 4C and S11A). Lastly, thermogravimetric analysis (TGA) revealed a decomposition temperature (*T*_d,5%_) of 246 °C, in agreement with traditional PHA susceptibility to thermal degradation (Figure S12A).^10^

Recognizing that cross-linking PHBU would impart higher stiffness to the brittle parent polymer, we considered strategies to enhance the mechanical performance while retaining the vitrimer dichotomy of melt processability and thermoset behavior. Conceivably, introducing the recently reported unsaturated *mcl*-*co*-*mcl* PHDU-6,^36^ a soft and amorphous elastomer (Figure 4D and Table S4, Figures S13, S14 for polymer characterization data), during the cross-linking stage presents the means for a two-component polymer system that could yield dynamic thermoset blends with a high degree of tunability in thermal and mechanical profiles. We posited that such a system could access a broad range of rubber-like performance profiles by utilizing unsaturated PHA materials with diverse properties, produced using our engineered *P. putida* strains through the feeding of various fatty acids. To this end, we conducted physical blending of the hard PHBU-5 with soft PHDU-6 at varying mass ratios of 3:1, 1:1, and 1:3, followed by cross-linking with dynamic boronic ester linkages. Initial analysis by scanning electron microscopy (SEM) at the cryo-fractured cross-section revealed a pool of phase-separated PHBU-5 and PHDU-6 in the typical form of droplet-style domains with average diameters between 0.6 and 13.9 μm buried in the host-polymer matrix (Figures 4E and S15–S17). Notably, a trend in reduced blend compatibility was observed with an increase in the PHDU-6 loading, likely caused by incompatibility with highly crystalline PHBU-5. Specifically, droplet size analysis revealed that increasing the PHDU-6 content from 25 to 50 to 75 wt % paralleled an increasing average droplet diameter of 1.7, 2.2, and 5.9 μm, respectively (Figures 4E and S18–S20).

It is well understood that the presence and corresponding size of droplets is a direct metric for blend performance as the low interfacial adhesion (high interfacial tension) between the two immiscible polymers sabotages interdomain entanglement.^48^ Specifically, larger droplets reflect poor compatibility and greater phase separation, while smaller droplets indicate a higher degree of compatibility. With this in mind, we used the usPHA pendent functionality to install dynamic cross-links as a compatibilization strategy. To this end, UV-photoinitiated thiol–ene click chemistry was performed on 3 g scales for the 3:1, 1:1, and 1:3 w/w usPHA blends in solution to covalently link the unsaturated pendent groups on both PHBU-5 and PHDU-6 with the dynamic boronic ester cross-linker, 2,2-(1,4-phenylene)-bis[4-thioethyl-1,3,2-dioxaborolane] (BE-X), capable of boronic ester metathesis for network reconfiguration (Figure 4D). The cross-linked blends are identified as PHBU_3_-*blend*-PHDU_1_, PHBU_1_-*blend*-PHDU_1_, and PHBU_1_-*blend*-PHBU_3_, where subscripts denote blend ratios by weight. Upon revisiting the dynamic thermoset blends with SEM, cross-sectional interfaces revealed homogenized surfaces in the complete absence of differentiable microdomains, signaling compatibilization (Figures 4E and S15–S20).

The resulting materials exhibited a gradient of thermal properties from semicrystalline (3:1 and 1:1) to fully amorphous (1:3) (Figures 4B and S10B–D). Successful installation of the dynamic cross-links was supported by DMA thermograms for each of the three blends, revealing extended thermomechanical performance windows in the rubbery plateau (Δ*E*′ ∼ 0) region beyond the ∼145 °C melt-failure of the virgin PHBU-5 parent polymer due to the associative nature of the boronic ester exchange retaining a constant cross-link density during metathesis events (−75 to 200 °C, 3 °C min^–1^, 30 μm, 1 Hz, Figures 4C and S11B–D).

More exciting, tensile stress/strain (5 mm min^–1^, ∼23 °C) revealed a wide range of thermoset performances dramatically outperforming the brittle parent PHBU-5 (Figure S9B–D). Specifically, we obtain a high modulus and stiff rubber (σ_B_ = 15.1 MPa, ε_B_ = 22%, *E* = 665 MPa) from the compatibilized 3:1 blend, a ductile polymer with intermediate modulus (σ_B_ = 6.6 MPa, ε_B_ = 94%, *E* = 70 MPa) from the 1:1 blend, and a soft elastomer (σ_B_ = 2.0 MPa, ε_B_ = 187%, *E* = 5.7 MPa) from the 1:3 blend (PHBU-5:PHDU-6) (Figure 4A and Table S5). Similar observations were made through the DMA thermograms for the 3:1, 1:1, and 1:3 blends, where a clear trend in decreasing *E*′_max_ of 4887, 3135, and 2941 MPa, respectively, is observed with decreasing loading of the more crystalline PHBU-5 (Figure 4C and Table S7).

### Dynamic Cross-Link Orthogonality by Elastic Recovery and Reprocessability

**Figure 5:**
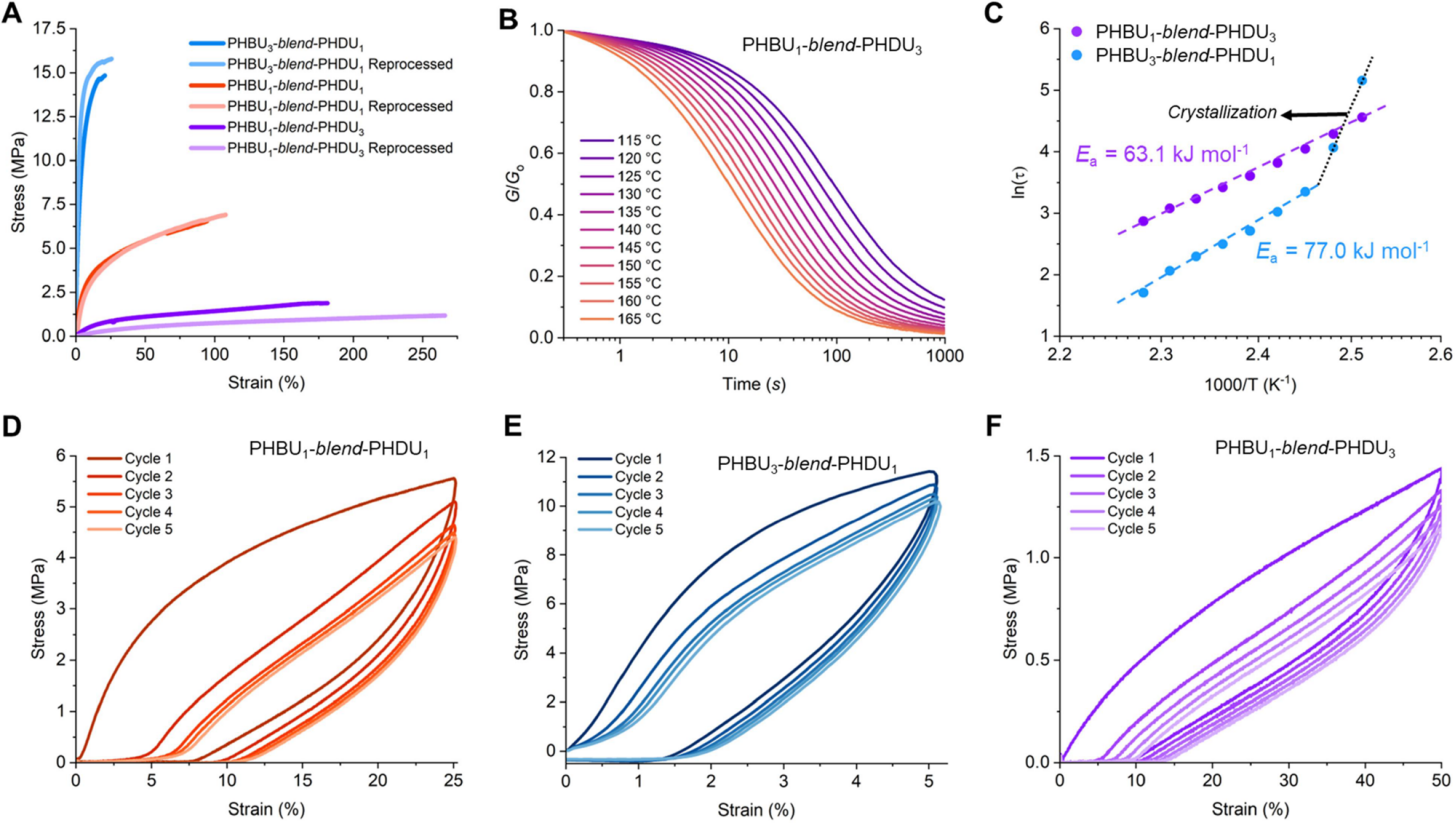
Elasticity and melt processability by dynamic cross-linking. (A) Representative averaged tensile stress/strain (5 mm min^–1^, ∼23 °C) overlays between virgin and reprocessed dynamic thermoset blends (see Figure S22 for complete data and Table S5 for values). (B) Normalized stress relaxation traces for PHBU_1_-*blend*-PHDU_3_ by shear rheology (115 to 165 °C, 5 °C increments, 5%, 1000 s, 0.1 s rise time). (C) Arrhenius relationships between temperature and relaxation time for PHBU_1_-*blend*-PHDU_3_ (purple) and PHBU_3_-*blend*-PHDU_1_ (blue), the latter of which demonstrates a deviation in slope upon crystallization. (D) Cyclic deformation experiments with firm plastic-like (PHBU_1_-*blend*-PHDU_1_), (E) hard rubber (PHBU_3_-*blend*-PHDU_1_), and (F) soft elastomer (PHBU_1_-*blend*-PHDU_3_) (50 mm min^–1^ up-ramp, 50 mm min^–1^ down-ramp, ×5, ∼23 °C).

The value of dynamic thermosets lies in the thermoset-like performance during operating conditions and thermoplastic-like melt reprocessability during reprocessing conditions. To demonstrate this, we first subjected each of the dynamic thermoset blends to a reprocessing cycle at sufficient temperatures (165 °C and 15 min) for full blend melting and thermal activation of dynamic boronic ester metathesis. As expected, scraps from the initial round of tensile analysis were reformed into homogeneous films from which renewed tensile bars were obtained and analyzed (Figures S21, S22 and Table S5). The reprocessed hard rubber PHBU_3_-*blend*-PHDU_1_ demonstrated a similar tensile profile to the original set in σ_B_ (15.1 MPa vs 15.8 MPa), ε_B_ (22 vs 26%), and *E* (665 vs 647 MPa) (Figure 5A). Performance retention was also observed in the reprocessed PHBU_1_-*blend*-PHDU_1_ sample, which overlays nearly identical to the virgin counterpart (Figure 5A). Surprisingly, we found that the soft elastomer PHBU_1_-*blend*-PHDU_3_ demonstrated significantly higher (+80%) average ε_B_ while exhibiting almost 2× reduction in ultimate σ_B_, indicating a possible change in the network architecture (Figure 5A, Table S5). The deviation in tensile performance may be accredited to boronic ester ambient hydrolysis susceptibility, thoroughly investigated in our previous PHA vitrimer systems relying on this specific boronic ester linkage.^36^ Due to the reduced crystallinity in these samples, water permeability may be higher, increasing the rate of hydrolysis. A higher rate of hydrolysis would thus lead to a reduction in the cross-link density and consequently the tensile toughness.

Thermoset melt reprocessability is afforded by the boronic ester’s ability to undergo thermally induced dynamic bond exchange. The thermodynamics of the exchange can be evaluated by stress relaxation experiments, where an activation energy (*E*_a_) for the exchange can be extracted as well as used to gauge implications for network healing. We conducted oscillatory rheology stress relaxation experiments on PHBU_1_-*blend*-PHDU_3_ and PHBU_3_-*blend*-PHDU_1_ to understand the impact of blend composition on these behaviors (Figures 5B and S23). In both cases, the dynamic blends exhibited stress relaxation times decreasing with higher temperatures. Interestingly, we found that despite the higher modulus of PHBU_3_-*blend*-PHDU_1_, the elastomeric PHBU_1_-*blend*-PHDU_3_ demonstrated a slower relaxation profile. We surmise that this may be due to the alkyl substituent length, where PHBU_1_-*blend*-PHDU_3_ has a significantly higher composition of sterically hindering dodecyl pendent chains. Conversely, transforming the stress relaxation to an Arrhenius plot (Figure 5C) yields an even more intriguing result where PHBU_1_-*blend*-PHDU_3_ has a lower *E*_a_ (63.7 kJ mol^–1^) for bond exchange than PHBU_3_-*blend*-PHDU_1_ (77.0 kJ mol^–1^), despite slower network relaxation times. This suggests that there is a valuable relationship between PHBU and PHDU pendant group sterics, *T*_g_, and dynamic bond exchange wherein ideal vitrimers with fast relaxation times and higher *E*_a_ barriers are obtained.

To examine elasticity, we subjected each blend to elastomer hysteresis analysis to observe the deformation recovery following cyclic stress loadings. Notably, the strain for deformation was varied between 5 and 50% in accordance with the ultimate strain obtained from initial tensile pulling tests. Following five load–unload cyclic ramps, the ultimate stress was retained at 89, 80, and 81% of the initial first cycle value on the 3:1 hard, 1:1 firm, and 1:3 soft cross-linked blends, respectively, highlighting moderate elasticity following rapid extension events (50 mm min^–1^ up-/down-ramp rate) (Figure 5D–F). Notably, we still observe some compounding loss in ultimate stress during terminal-end strain loadings as a result of crystalline region disruption. Overall, we credit the high tunability of these thermomechanical profiles to the thermal properties, where higher crystallinity lends itself to higher strength, lower ductility, and increased consequences for elastic recovery.

### Biodegradability of Virgin and Cross-Linked Materials

**Figure 6:**
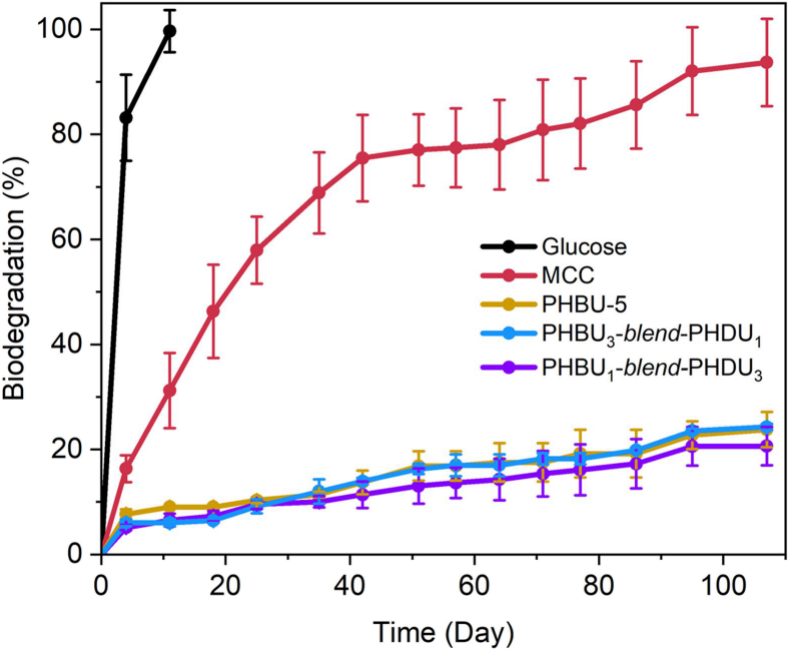
Biodegradability evaluation in freshwater biodegradation experiment. Freshwater biodegradation of PHBU-5, PHBU_1_-*blend*-PHDU_3_, and PHBU_3_-*blend*-PHDU_1_ in freshwater environments. Results over a 107-day period of aerobic freshwater degradation (ISO 14851)^49^ for PHA dynamic thermoset blends relative to a microcrystalline cellulose (MCC) and a glucose control.

To assess the biodegradability of the cross-linked PHA copolymer films in comparison with non-cross-linked PHBU-5, biodegradation studies of the 1:3 and 3:1 blends in a freshwater environment were conducted, adhering to the ISO 14851 standard test.^49^ In this context, PHBU-5, PHBU_1_-*blend*-PHDU_3_, and PHBU_3_-*blend*-PHDU_1_ reached 23.8 ± 3.4, 20.6 ± 3.7, and 24.3 ± 0.6% biodegradation levels, respectively, in 107 days (Figure 6). To estimate the lifetime of these films in freshwater environments, we applied first-order kinetics, which suggests that PHBU-5 and its cross-linked blends are expected to achieve 90% biodegradation between approximately 1000 and 1300 days (Table S9). Statistical analysis revealed no significant difference in their biodegradation rates in this freshwater environment (*p* > 0.05), suggesting that the cross-linking using BE does not affect the biodegradability of materials. Notably, all three films exhibit a dual-stage degradation pattern, with accelerated biodegradation observed around day 20 and another spike around day 80. This feature is likely related to the degradation of different segments (e.g., amorphous vs crystalline, short chain vs long chain) or individual components (e.g., PHBU or PHDU) of the blends. Further evidence for partial biodegradation is the reduction in molar mass of the linear parent polymer PHBU-5 before and after the biodegradation experiment, which decreased by 15%, from 124 to 107 kg mol^–1^ (Figure S24). In summary, these findings show that the cross-linked blends have similar degradation profiles compared to the parent polymers.

## Discussion

In this report, we demonstrated a feasible, two-component platform for dynamic thermoset blend production encompassing a broad range of thermal and mechanical profiles from biologically produced PHAs. The unsaturated units in PHBU and PHDU were derived from 10-undecenoic acid, primarily synthesized via the pyrolysis of ricinoleic acid, the main fatty acid in castor oil. Notably, PHA containing other unsaturated monomer units, such as 3-hydroxy-5-dodecenoate (3H5DD), has been produced in *P. putida* grown on unrelated carbon sources like glucose^50^ and gluconate^51^ via the FAB pathway. In our study, the FAB pathway was deliberately blocked to prevent its contribution to PHA synthesis (Figure 2), enabling the precise control of PHA composition for material characterization. Interestingly, the presence of unsaturated units, such as 3H5DD, may have been overlooked for decades due to limitations in conventional GC/MS and NMR spectroscopy workflows.^51^ Recent advancements, such as thiomethyl pretreatment, now facilitate easier detection of these units.^51^ Investigating the *in vivo* generation of unsaturated fatty acids remains a promising direction for future research.

Blending *scl*- and *mcl*-PHAs has been explored to mitigate the brittle nature of ubiquitous P3HB and other *scl*-PHAs. For example, blending elastomeric *mcl*-PHA poly(3-hydroxy-octanoate) with P3HB led to enhanced mechanical performance when the P3HB loading <20%, obtaining ductility values of ∼200%, which is similar to our thermoset PHBU_1_-*blend*-PHDU_3_.^52^ However, these polymers showed high immiscibility accredited to stark differences in crystallinity and *T*_m_ at all ranges of P3HB incorporations. Thus, compatibility between an *scl* and *mcl* PHA during blending is highly dependent on the ability to cocrystallize, which is challenging due to the large difference in *T*_m_ values (*T*_m_*scl* PHA ≪ *T*_m_*mcl* PHA).^53^ In agreement, the *scl*-PHA in this study, PHBU-5, which is similar in brittleness to P3HB, also displays a lack of inherent compatibility with amorphous PHDU-6. This is highlighted in Figure 4E and is a result of the differences in crystallinity between the polymers, wherein increased loadings of PHDU yield increased immiscibility. These cocrystallization considerations are no longer an impedance upon installment of dynamic cross-links, as evidenced by the compatibilization across all PHBU-*blend*-PHDU compositions. Furthermore, thermoplastic blends have performance limitations when it comes to elasticity. In Figure 5D–F, the hysteresis curves revealed moderate elastic recovery (80–90% stress), notably beyond the capacity for thermoplastic materials, which lose most of the performance following the yield point. Crystallinity can be reduced or ultimately eliminated by increasing the loading of PHDU or the boronic ester cross-linker to obtain elastomer properties, as it is well-known that installing cross-linkers effectively reduces crystalline domain formation by disrupting the chain packaging capability.^54,55^

To the best of our knowledge, there are no examples of PHA-based blends that can serve as thermosets and meet the same degree of tunability and reprocessability at the EoL as with thermoplastics. As a result of the scarcity of literature reports on similar material design, we compare our PHA-based blends’ performance to those of existing commercial thermosets. We find that our most elastic substrate, PHBU_1_-*blend*-PHDU_3_, offers similar performance in tensile strength in Figure 4A for a recyclable replacement to untreated (nonvulcanized) nitrile (acrylonitrile-*co*-butadiene, NBR), styrene–butadiene (SBR), and natural (isoprene) rubber, although increasing PHDU loading is likely required to achieve analogous (200–600%) ductility. Conversely, vulcanized rubbers exhibit tensile moduli similar to that of our more crystalline dynamic thermoset PHBU_3_-*blend*-PHDU_1_ (σ_B_ = 15.1 MPa). Through compatibilizing thermoset blends with distinctly dynamic linkages, we demonstrate the ability to access tensile profiles from elastomeric to high-modulus thermoset responses with simple changes in the blend ratio. Although examples of solution-based processes for rubbers exist at large scales, such as anionic polymerization of SBR,^56^ in future, we aim to accomplish blending and compatibilization under a single melt processing step.

Finally, biodegradation tests are imperative for new materials as they indicate whether a material will persist if it ends up in the natural environment. When ultimately formulating PHA-based thermosets for specific applications, it is important that additives and modifiers do not compromise the biodegradability. An example of microbially produced *mcl*-PHA blended with thermoset materials such as natural, butadiene, and nitrile rubbers demonstrated, perhaps unsurprisingly, that biodegradability is only achieved in the PHA component.^38^ Another report found that the biodegradation rate of peroxide-mediated cross-linked PHA was reduced by half, convincingly as a result of irreversible C–C cross-linking and structural disordering.^24^ Contrarily, by constructing blends based only on PHAs, over 20% biodegradation is achieved at 107 days (Figure 6) and is projected to fully decompose in under 4 years. Despite the cross-linked architecture, biodegradability of the PHBU-*blend*-PHDU systems is equivalent to the PHBU-5 parent polymer. We suspect that this may be due to a range of factors including similar film surfaces and hydrolytic instability of boronic esters.

Our study suggests future research in producing PHA thermosets with customized profiles. Notably, the broad substrate polymerization capabilities of PhaC_61-3_-S325T, Q481K, covering precursors C_4_, C_10_, C_11_, and C_12_ (Figure 3A), suggest the potential use of strain LC059 as a platform to produce a diversity of *scl*-*co*-*mcl* PHA copolymers comprising multiple monomers through the feeding of related fatty acids. Future designs for PHA thermosets could exploit different reversible chemistries through the alkene functionality, such as epoxy-acid chemistry, to yield polyester CANs. All-PHA dynamic thermoset blends are thus a promising solution to compatible, tunable rubbers while preserving biodegradability and reprocessability at the EoL.

## Conclusions

We present a two-component platform for renewable and biodegradable dynamic thermoset blends with a high degree of thermomechanical tunability from the simple dialing of otherwise immiscible microbially produced polymer ratios. We characterized the biosynthesis of the targeted PHA materials, showcasing their predictability, reproducibility, and scalability across different production means, from shake flasks to bioreactors. Physical blends of various microbial-produced PHA copolymers can be compatibilized through chemical cross-linking via the thiol–ene click reaction using dithiol boronic ester, producing hard, medium, and soft rubber-like materials. Furthermore, our biodegradability tests confirm that our modifications did not significantly compromise the biodegradability of PHA copolymers. Overall, the strategy of bioproduction, followed by blending and chemical modification, offers a promising approach for producing a wide range of sustainable thermoset materials based on PHAs.

## Experimental Section

### Strain Engineering

Plasmid pLC007 was transformed into strain LC039 via electroporation. The electroporation method was modified from a previous study.^57^ Strain LC039 was inoculated from a glycerol stock into 14 mL culturing tubes with 5 mL of LB medium and were incubated overnight in an incubator at 30 °C and 225 rpm. To prepare for electroporation, 1 mL of the overnight culture was harvested by centrifugation at 6000 rpm for 1 min in an Eppendorf 5424R benchtop centrifuge. The supernatants were carefully removed, and the cell pellets were subjected to two successive washes: first with 1 mL of 300 mM sucrose and then with 500 μL of 300 mM sucrose. After washing, the cell pellets were resuspended in 50 μL of 300 mM sucrose, resulting in a final resuspended cell volume of approximately 100 μL, taking into account any residual sucrose in the pellets. Next, 1 μg of plasmid DNA was gently mixed with the resuspended cells. This cell–plasmid mixture was then transferred into a 0.1 cm electroporation cuvette (BioRad) and subjected to electroporation using the following parameters: 1.6 kV, 25 μF, 200 ohms. Immediately after electroporation, 950 μL of LB medium was added to the cuvette. The LB medium containing the electroporated cells was then transferred into a 1.5 mL Eppendorf tube, which was securely attached to the surface of an incubator. The culture was shaken at 30 °C for 2 h. Following the incubation, the cells were centrifuged in an Eppendorf 5424R benchtop centrifuge at 6000 rpm for 1 min. Most of the medium was removed, leaving approximately 100 μL behind. The remaining culture was resuspended and spread onto LB agar plates containing 50 μg mL^–1^ kanamycin (LB-Kan plates). Transformants were then restreaked on a separate LB-Kan plate to isolate single colonies. These single colonies were utilized in the subsequent SacB counter-selection process.^57^

### Shake Flask Experiments

The seed train for strain LC059 was conducted as follows. Strain LC059 was inoculated from glycerol stock into 14 mL culturing tubes containing 5 mL of LB medium and incubated overnight at 30 °C and 225 rpm (*initial seed cultures*). The initial seed cultures were then inoculated into 125 mL baffled flasks containing 25 mL of LB medium, with 1% inoculating volume (*second seed cultures*). The second seed cultures were incubated at 30 °C and 225 rpm for around 8 h, reaching an OD_600_ around 4. To prepare the medium for PHBU production in shake flask experiments, LB broth (Miller) powder was first added into 2.8 L flasks, followed by the addition of appropriate amounts of sodium butyrate and 10-undecenoic acid. Finally, 4 g L^–1^ Brij-35 detergent was added to the mixtures (to enhance the solubility of the fatty acids) and the entire content was thoroughly mixed and subsequently autoclaved. In the case of the shake flask experiments using 500 mL flasks, 100 mL of the autoclaved medium was transferred from the 2.8 L flasks into each of the 500 mL flasks. The second seed cultures mentioned above were then inoculated into either 500 mL baffled flasks with 100 mL of the prepared medium or 2.8 L baffled flasks with 500 mL of the prepared medium to an initial OD_600_ of 0.05. All shake flask experiments were run for around 66 h. After completion of the experiments, the final cell cultures were subjected to centrifugation at 10,000 rpm for 10 min. The supernatants were discarded, and the cell pellets were washed with equal volumes of Milli-Q water and 70% ethanol, separately. These washed cell pellets were then placed in a −80 °C freezer for a minimum of 3 h, followed by freeze-drying for at least 24 h.

### Sodium Butyrate Toxicity Test

The seed culture was prepared as follows. *P. putida* LC059 was scrapped from a glycerol stock, inoculated in 250 mL baffled flasks containing 50 mL of the LB-Miller medium, and incubated overnight (16 h) at 30 °C and 225 rpm. The cells were harvested by centrifugation at 5000 rpm for 10 min and then resuspended in a modified M9 medium. This medium consisted of 13.56 g L^–1^ Na_2_HPO_4_, 6 g L^–1^ KH_2_PO_4_, 1 g L^–1^ NaCl, 2 g L^–1^ (NH_4_)_2_SO_4_, 2 mL L^–1^ of 1 M MgSO_4_, 0.1 mL of 1 M CaCl_2_, and 1 mL L^–1^ of 1 mM FeSO_4_. M9 was supplemented with glucose (9 g L^–1^ = 50 mM). The resuspended cells were then inoculated in 96-well plates containing modified M9 media (200 μL) with different concentrations of sodium butyrate (1, 2.5, and 5 g L^–1^) at an initial OD_600_ of 0.2. The plates were incubated at 30 °C in the high-speed mode for 48 h using LogPhase 600 (BioTek, Inc.). Assays at the different sodium butyrate concentrations were conducted in triplicate.

### PHBU Production in Bioreactors

The strain utilized for PHBU production in bioreactors was *P. putida* strain LC059. The preparation of the seed culture was conducted as follows. The surface of the bacterial glycerol stock was scrapped and inoculated in 250 mL baffled flasks containing 50 mL of LB-Miller media. The seed was incubated for 16 h at 30 °C and 225 rpm (*initial seed culture*). Then, the cells were inoculated in 1 L baffled flasks containing 200 mL of LB-Miller media at an initial OD_600_ of 0.2 (*second seed culture*). Once the cells reached an OD_600_ of 2, the cells were harvested by centrifugation at 5000 rpm for 10 min and resuspended in 5 mL of modified M9 medium for further inoculation in 3 L bioreactors (Applikon) at an initial OD_600_ of 0.2. PHBU production in bioreactors was conducted using a modified minimal M9 medium in the fed-batch mode. The volume of the media in the batch phase was 1.2 L, and the modified M9 medium consisted of 13.56 g L^–1^ Na_2_HPO_4_, 6 g L^–1^ KH_2_PO_4_, 1 g L^–1^ NaCl, 2 g L^–1^ (NH_4_)_2_SO_4_, 2 mL L^–1^ of 1 M MgSO_4_, 0.1 mL L^–1^ of 1 M CaCl_2_, 1 mL L^–1^ of 18 mM FeSO_4_, and 3 g L^–1^ glucose. The bioreactors were controlled at 30 °C and pH 7 with an airflow rate of 1 vvm. The pH was automatically controlled by the addition of 4 N NaOH. The initial agitation was set at 350 rpm, and the initial DO was 100%. When the DO decreased to 30%, the DO was automatically controlled by changes in the agitation speed. The fed-batch phase initiated when glucose was depleted from the batch medium, and the DO level increased up to 75%. Three independent feeding solutions were prepared: (1) a solution with 500 g L^–1^ glucose and 100 g L^–1^ (NH_4_)_2_SO_4_ adjusted to pH 7 with 4 N NaOH, (2) a solution of 100 g L^–1^ sodium butyrate, and (3) a solution of 10-undecenoic acid (98% purity, Sigma). Solution (1) was added following a DO-stat fed-batch strategy. This recipe was programmed to add 1 mM glucose every time the DO reached a level of 75%, and agitation was manually controlled. Solutions (2) and (3) were manually added to the bioreactors at different times during the cultivations to achieve a sodium butyrate concentration of 2 g L^–1^ and a 10-undecenoic acid concentration of 0.16 g L^–1^. Antifoam 204 (Sigma-Aldrich) was added as necessary. Samples were collected during cultivation in bioreactors to measure the optical density at 600 nm (OD_600_), glucose, and butyrate. It is worth noting that OD_600_ is not an indicator of bacterial growth because the accumulation of PHBU also increases the value of the optical density. Glucose was analyzed in a glucose analyzer (YSI 2500 Biochemistry Analyzer). At the end of the cultivation, cells were collected from three independent bioreactors by centrifugation (5000 rpm, 10 min), washed twice with water, and utilized for CDW quantification and for downstream product purification and quantification. 1278 mL was the final volume in the bioreactors.

### Chromatographic Analysis

The methodologies employed for the chromatographic analysis of PHA samples and fatty acids remained consistent with our prior investigation,^36^ except for butyric acid. In the case of butyric acid, analysis utilized an Agilent Technologies 1260 series high-performance liquid chromatography system with a refractive index detector (RID) used for analyte detection and an Aminex HPX-87H (300 × 7.8 mm, Biorad Laboratories) column. Samples and standards were injected on the column at a volume of 6 μL. The column compartment and RID were maintained at 55 °C, and chromatographic separation was achieved using a mobile phase of 0.01 N sulfuric acid with an isocratic flow rate of 0.6 mL min^–1^ for a runtime of 27 min. The calibration curve for butyric acid was assessed at concentrations from 0.5 to 30.0 g L^–1^ with a minimum of five calibration standards used, resulting in a curve with an *R*^2^ coefficient of 0.995 or better. A calibration verification standard was analyzed every 10–15 samples to ensure the integrity of the initial calibration throughout the analysis run.

### Polymer Isolation and Purification

Lyophilized biomass was stirred vigorously in CHCl_3_ for 24 h. The mixture was filtered through a Chemrus disposable 10 μm pore size filter funnel and then through a 0.2 μm PTFE filter frit. The polymer solution was concentrated on the rotary evaporator until viscous and then precipitated from cold ethanol. The polymer was recovered by filtration and dried in a vacuum oven at 40 °C for 24 h.

### Blending and Thiol–Ene Click Cross-Linking Compatibilization

A 250 mL round-bottom flask was charged with PHBU-5, PHDU-6, and Bis-BE-SH (5 equiv of SH:alkene) in a glovebox. DMPA (0.5 wt % relative to polymer) and degassed, anhydrous, inhibitor-free DCM (150 mL) were added under a positive flow of nitrogen on a Schlenk line. The flask was placed in an air-cooled photoreactor (365 nm LED light) and irradiated for 2 h. After the reaction, most of the DCM was removed in vacuo, and the mixture was precipitated in anhydrous methanol. The polymer was collected and dried in a vacuum oven at 40 °C for 24 h.

### Biodegradation Testing

CHN elemental analysis was conducted to quantify the total organic carbon and hydrogen in the samples employed for biodegradation assessment (Table S8). The biodegradability of polymer samples in a freshwater environment was assessed following the ISO 14851 methodology. Polymer film samples, including PHBU-5, PHBU_1_-*blend*-PHDU_3_, and PHBU_3_-*blend*-PHDU_1_ (approximately 4 mm × 3 mm × 1 mm in size), were tested in triplicates within 300 mL biological oxygen demand (BOD) glass bottles (VWR International). In each BOD bottle, activated sludge from a wastewater treatment plant (Lemont, IL, USA) was combined with 200 mL of aqueous medium, which was composed of the following components (in mg L^–1^): KH_2_PO_4_, 85; K_2_HPO_4_, 217.5; Na_2_HPO_4_, 334; NH_4_Cl, 15; MgSO_4_·7H_2_O, 22.5; CaCl_2_·2H_2_O, 36.4; and FeCl_3_·6H_2_O, 0.25. The total solid from the sludge was 60 mg L^–1^. For each of the triplicate test bottles of polymer samples, PHBU-5, PHBU_1_-*blend*-PHDU_3_, and PHBU_3_-*blend*-PHDU_1_ film samples (approximately 4 mm × 3 mm × 1 mm size) were added to the BOD bottles. The total organic carbon from polymer samples (PHBU-5, PHBU_1_-*blend*-PHDU_3_, and PHBU_3_-*blend*-PHDU_1_) added to every test bottle was 9.0 mg. Three blank test bottles with no additional carbon content other than the aqueous medium and three positive control bottles with 9.0 mg of total organic carbon content from d-glucose (Fisher Scientific, granular powder) and cellulose (microcrystalline, particle size 0.05 mm, Acros Organics) were also set up as positive controls. All bottles were incubated in a New Brunswick Scientific incubator shaker (Eppendorf, model I-24) at 25 °C and 150 rpm. BOD was determined by measuring oxygen consumption using a pH/RDO/DO meter (Thermo Fisher Scientific, model Orion Star A216) based on the conditions listed in the ISO 14851 standard method. The percentage biodegradability of the sample was calculated as
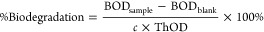
1The observed values of BOD_sample_ and BOD_blank_ were recorded for the sample and the blank bioreactor, respectively. The amount of sample added, denoted as “*c*”, was carefully measured. ThOD, representing the theoretical oxygen demand value of the sample, was calculated based on the chemical formula, assuming complete oxidation of the polymer sample. It was required by the protocol that the positive control (glucose) reach a biodegradation level of 60% at the conclusion of the test and that the standard deviation of each sample be less than 20% of the mean.

### Estimation of End of Lifetime

A first-order kinetic model was employed to calculate the biodegradation rate and predict the lifespan of the polymer samples in a freshwater environment.
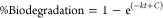
2Equation 2 was utilized to create a plot of percentage biodegradation against time. Within the equation, *k* represents the rate constant, *t* denotes the reaction time, and *C* is a constant from the plot. Table S9 provides a summary of the rate constants and estimated times to reach 90% biodegradation in a freshwater environment, and *R*^2^ values indicate the fitness of the model.

See the Supporting Information for materials and polymer characterization instrumentation and methods.

## Supporting Information Available

Source data for each figure, where applicable (XLSX)
Materials, NMR spectra, tensile data, DSC and TGA graphs, DMA data, SEM images (PDF)

## Notes

R.W.C., G.R., R.M.C., N.A.R., and G.T.B. are listed as coinventors on US patent application US20240239956A1.
