# Supplementary material for: Tunable and Degradable Dynamic Thermosets from Compatibilized Polyhydroxyalkanoate Blends
Source: ACS Sustain Chem Eng. 2025 Feb 27;13(9):3817–29. doi: 10.1021/acssuschemeng.5c00943 (PMC11898171; doi:10.1021/acssuschemeng.5c00943)
Supplement: Supplementary file 2 — sc5c00943_si_002.pdf [file sc5c00943_si_002.pdf]

Supporting Information

Tunable and degradable dynamic thermosets from compatibilized polyhydroxyalkanoate blends

Chen Ling,[a,b,‡] Ryan W. Clarke,[a,c,‡] Gloria Rosetto,[a,‡] Shu Xu,[d,e] Robin M. Cywar,[a] Dong Hyun Kim,[a] Levi J. Hamernik,[a] Stefan J. Haugen,[a,c] William E. Michener,[a,b,c] Sean P. Woodworth,[a,c] Torrey M. Lind,[a,b] Kelsey J. Ramirez,[a,b,c] Meltem Urgun-Demirtas,[d] Davinia Salvachúa,[a,b] Christopher W. Johnson,[a,b] Nicholas A. Rorrer,[a,c,*] and Gregg T. Beckham[a,b,c,*]

a. Renewable Resources and Enabling Sciences Center, National Renewable Energy Laboratory, Golden CO, 80401, USA
b. Agile BioFoundry, Emeryville, CA 94608, USA
c. BOTTLE Consortium, Golden, CO 80401 USA
d. Applied Materials Division, Argonne National Laboratory, Lemont, IL 60439 USA
e. Northwestern Argonne Institute of Science & Engineering, Evanston, IL 60208 USA

‡ Denotes equal contribution
* Corresponding author email: nicholas.rorrer@nrel.gov; gregg.beckham@nrel.gov

Table of Contents

Materials and Methods ***S3***

Materials **S3**

Polymer characterization methods **S3**

Supplementary tables ***S5***

Table S1. Construction details for plasmids used for gene replacements in this study. **S5**

Table S2. Strain construction details **S5**

Table S3. Oligonucleotides and dsBlock DNA fragments used in this study. **S5**

Table S4. Polymer properties of PHDU-6 used in this study **S6**

Table S5. Average tensile stress/strain values for each reported sample. **S7**

Table S6. Tabulated DSC data of PHBU-5 and its blends **S7**

Table S7. Tabulated DMA storage modulus (*E*') values for PHBU and each blend at low temperature (E'_{max}, −75 °C) and room temperature (E'_{RT}, 23 °C). **S7**

Table S8. CHN Elemental Analysis results for samples used for biodegradation test. **S7**

Table S9. Summary of estimated lifetime of PHBU film samples using first order kinetic model. **S8**

Supplementary figures ***S9***

Figure S1. Shake flasks experiment in 500 mL flasks. **S10**

Figure S2. Profiles of shake flasks experiment in 2.8L flasks. **S10**

Figure S3. Dissolved oxygen (DO) and agitation profiles **S11**

, panels A and B represent profiles from two independent bioreactors. The data was collected during PHBU production in modified minimal M9 medium and DO-stat fed-batch mode. Black arrows indicate the time at which sodium butyrate (2 g L^{-1}) and 10-undecenoic acid (0.16 g L^{-1}) were added in the bioreactors. These profiles correspond to the bioreactor results shown in Fig. 3D. **S11**

Figure S5. ^{1}H NMR ($CDCl_3$, 400 MHz) spectrum of PHBU-5. ..... **S13**

Figure S6. $^{13}C\{^{1}H\}$ NMR ($CDCl_3$, 101 MHz) spectrum of PHBU-5. ..... **S14**

Figure S7. FTIR spectrum of PHBU-5. .... **S15**

Figure S8. $CHCl_3$ GPC trace of PHBU-5. .... **S16**

Figure S9. Individual stress/strain curves (~23 °C, 5 mm min^{-1}) for A) PHBU-5, B) $PHBU_3$-*blend*-$PHDU_1$ C) $PHBU_1$-*blend*-$PHDU_1$ and D) $PHBU_1$-*blend*-$PHDU_3$ **S17**

Figure S10. DSC traces **S18**

Figure S11. Individual DMA thermograms **S19**

Figure S12. TGA graphs **S20**

Figure S13. $CHCl_3$ GPC trace of PHDU-6. .... **S20**

Figure S14. ^{1}H NMR spectrum ($CDCl_3$, 400 MHz) of PHDU-6. .... **S21**

Figure S15. Additional SEM images of film cross-sections for virgin (top) and compatibilized (bottom) $PHBU_1$-*blend*-$PHDU_3$ **S21**

Figure S16. Additional SEM images of film cross-sections for virgin (top) and compatibilized (bottom) $PHBU_1$-*blend*-$PHDU_1$ **S22**

Figure S17. Additional SEM images of film cross-sections for virgin (top) and compatibilized (bottom) $PHBU_3$-*blend*-$PHDU_1$ **S22**

Figure S18. Droplet diameter analysis for $PHBU_1$-*blend*-$PHDU_3$ cross-sectional images. **S23**

Figure S19. Droplet diameter analysis for $PHBU_1$-*blend*-$PHDU_1$ cross-sectional images. **S24**

Figure S20. Droplet diameter analysis for $PHBU_3$-*blend*-$PHDU_1$ cross-sectional images. **S24**

Figure S21. Digital images for (top) virgin $PHBU_3$-*blend*-$PHDU_1$ (left), $PHBU_3$-*blend*-$PHDU_1$ (middle), and $PHBU_3$-*blend*-$PHDU_1$ (right) tensile scraps, and (bottom) corresponding reprocessed films (165 °C, 5 min). **S25**

Figure S22. Individual tensile stress/strain (~23 °C, 5 mm min^{-1}) curves for reprocessed A) $PHBU_3$-*blend*-$PHDU_1$, B) $PHBU_1$-*blend*-$PHDU_1$, and C) $PHBU_1$-*blend*-$PHDU_3$. **S25**

Figure S23. Normalized stress relaxation traces for $PHBU_3$-*blend*-$PHDU_1$ by shear rheology **S26**

Figure S24. Overlay of GPC traces of PHBU-5 before and after freshwater degradation for 90 days. **S26**

References ***S27***

Materials and Methods

Materials

Sodium butyrate, 10-undecenoic acid, and Miller's Lysogeny Broth (LB), chloroform (HPLC grade, ethanol inhibitor), methanol (anhydrous Sure/Seal^{TM}), dichloromethane (anhydrous Sure/Seal^{TM}), 2,2-dimethoxy-2-phenylacetophenone (DMPA), and chloroform-*d* (99.8 atom% D, contains 0.03% (v/v) TMS) were purchased from Sigma Aldrich and used as received. Surfactant Brij-35 was purchased from Santa Cruz Biotechnology. D-glucose (granular powder) was purchased from Fischer Scientific. Cellulose (microcrystalline, particle size 0.05 mm) was purchased from Acros Organics. Bis-BE-SH was synthesized according to a literature procedure.[1]

Polymer characterization methods

Nuclear Magnetic Resonance (NMR) Spectroscopy. ^{1}H and ^{13}C spectra of linear polymers were recorded on a 400 MHz Bruker instrument (FT 400 MHz, ^{1}H; 101 MHz, ^{13}C) at ambient temperature. Chemical shifts were referenced to internal solvent resonances and reported as parts per million (ppm) relative to tetramethylsilane. A delay (d1) time of 30 seconds was required for accurate quantification of mol% U in PHBU.

Thermogravimetric Analysis (TGA) in to investigate thermos decomposition of PHBU. TGA was performed using a TA Instruments TGA-5500 at a heating rate of 20 °C min^{-1} under 50 mL min^{-1} of N_2 gas. The onset degradation temperature was determined using Trios software, defined at 5% mass loss ($T_{d,5}$). Derivative curves were generated automatically by Trios software.

Differential Scanning Calorimetry (DSC). DSC studies were conducted using a TA Instruments DSC-Q2000 or DSC-25 and analyzed with Trios software. Typical polymer samples (~5 mg) were run between -75 and 200 °C at heating and cooling ramp rates of 10 °C min^{-1}, from which second heating scans are reported. Exotherms are in the upward direction for all DSC figures.

Gel Permeation Chromatography (GPC). The columns consisted of three Agilent PLgel 10 µm Mixed-B LS 300 × 7.5 mm columns and a matching guard column attached in series. HPLC grade chloroform, stabilized with ethanol (Sigma Aldrich) was used as the mobile phase. The samples were dissolved in chloroform at a concentration of ~5 mg mL^{-1}. The sample was then filtered through a 2 µm filter directly into a 1.5 mL GC vial. The operating conditions included using chloroform as the mobile phase, a flow rate of 1.0 mL min^{-1}, column oven temperature set to 40 °C, and a sample injection of 100 µL. Detectors consisted of a miniDawn Multi-Angle Light Scattering detector (Wyatt Technology) used in combination with a Optilab Differential Refractive Index detector (Wyatt Technology). Wyatt Technologies Astra Software was used to analyze data.

Tensile tests. Compression molded films were prepared by placing bulk polymer (~3 g) into a stainless-steel rectangular frame and sandwiched between two aluminum sheets each lined with Teflon paper. Films were melt pressed for 10 – 15 minutes at 160 °C using a Carver heating hydraulic press and the resulting films were cut using an ASTM D638 type-V dog-bone–shaped die. Tensile testing was conducted on an Instron 5900 tensiometer equipped with a 1-kN load cell. A strain rate of 5 mm min^{-1} was used at ambient temperature using a minimum of 3 specimens. Data was automatically generated and processed using Bluehill Universal Software. Cyclic tension experiments (hysteresis) were constructed in the TestProfiler software using 50 mm min^{-1} strain rate and back to specified strains for 5 cycles.

Dynamic mechanical analysis. Rectangular polymer samples (~18 mm × ~3 mm × ~0.3 mm) were loaded into film tension clamps stationed on a Q850 DMA under steady flow of N_2 (TA Instruments). Sub-ambient (-75 °C)

temperatures were probed by an ACS-3 (Air Chiller System). Temperature-ramp frequency sweeps were run between -75 ˚C and 200 ˚C (3 ˚C min^{-1}) at an oscillating strain of 15 micrometers (1 Hz). Maximum working storage modulus (E'_{max}) was recorded as the highest obtained value (-75 ˚C), room temperature storage modulus (E'_{RT}) was recorded at the value at 23 ˚C, and the tan δ (E''/E') peaks were used to corroborate film T_g or T_m with those from DSC. Data collection and analysis were performed on the publicly available Trios software (TA Instruments).

Oscillatory Rheology. Stress relaxation experiments were performed on a Discovery Series Hybrid 20 (DHR-20) Rheometer (TA Instruments). Polymer films with a thickness of ~400 μm were loaded onto electrically-heated, disposable 25 mm parallel plate geometries under N_2 (30 psi) gas flow. Samples were equilibrated at specified temperatures for 5 min, following which a 5% strain was applied with a 0.1 s rise time and maintained for 1000 seconds. Stress relaxation was conducted between 115 °C and 170 °C in increments of 5 °C . Raw stress relaxation curves were transformed to normalized modulus plots (Go = G0/G0max) and the characteristic relaxation time (τ) was defined as the time to achieve a normalized modulus of 1/e (~0.37), in accordance with the Maxwell model. An Arrhenius relationship was employed to determine the apparent activation energy.

Scanning electron microscopy. Scanning electron microscopy (SEM) imaging was employed for the cross-sectional analysis of PHBU-*blend*-PHDU thin films and carried out on a Hitachi S-4800 High Resolution scanning electron microscope in low- and high-magnification modes at electron voltage = 10 kV, current = ~7 amps, and working distance = 4 mm. Prior to imaging, samples were cryo-fractured in liquid nitrogen to obtain cross-section pieces, which were then coated with a thin layer (10 nm) of Ir using a Cressington plasma sputter-coater (208-HR) under inert (Ar) atmosphere and mounted on an aluminum stage with double-sided C and Cu tapes. Image processing was conducted via publicly available software ImageJ2 (NIH).

Supplementary tables

Table S1. Construction details for plasmids used for gene replacements in this study.

Plasmid	Utility	Construction details
**pK18sB**	As backbone vector to build plasmid pCJ193.	Jayakody et al., 2018[4]
**pCJ193**	As backbone vector to build plasmid pLC007.	The upstream and downstream targeting regions for *phaC$_1$:C$_2$:Z$_1$* were amplified from gDNA of *P. putida* KT2440 using oGB343/oCJ746 and oCJ747/oCJ748, respectively. These PCR products and the gBlock: CJ_phaC1437 were assembled with pK18sB digested with EcoRI and HinDIII. The resulting vector was transformed into NEB 5-alpha F'*Iq E. coli*. This clone was confirmed by diagnostic digest with EagI and sequencing.
**pLC007**	Replacement of the native *phaC$_1$C$_2$Z* operon in *P. putida* KT2440 with engineered PHA synthase *phaC$_{61\text{-}3}$*-S325T, Q481K derived from *Pseudomonas sp.* 61-3.	The *phaC$_{61\text{-}3}$*-S325T, Q481K sequence was synthesized as gBlock by IDT, and was codon optimized for *P. putida* KT2440 using the IDT codon optimization tool. Plasmid backbone was amplified from plasmid pCJ193 using primers oLC0059 and oLC0060. The gBlock was diluted and assembled into backbone using NEBuilder® HiFi DNA Assembly Master Mix (NEB). The resulting product was transformed into NEB 5-alpha F'I^q (NEB) competent cells and plated on LB agar plates with 50 µg mL^{-1} kanamycin. Plasmids were then extracted and sent for sequencing.

Table S2. Strain construction details

Strain	Genotype	Construction details
**LC039**	*P. putida* KT2440 Δ*fadAB* ΔPP_2134 ΔPP_2135 Δ*fadBx*1 Δ*fadAx* Δ*fadBx*2 ΔPP_2218 Δ*phaG* ΔPP_2047:PP_2051 Δ*phaC$_1$phaZphaC$_2$*:*phaC*$_{61\text{-}3\text{-codon optimized}}$	LC039 derivative strain, the *phaC$_1$phaZphaC$_2$* operon was replaced with *phaC*$_{61\text{-}3\text{\_codon optimized}}$ using pLC007. The replacement was confirmed by amplification of a 3540 bp product in colony PCRs with oLC0099 and oLC0100.

Table S3. Oligonucleotides and dsBlock DNA fragments used in this study.

Name	Sequence (5'-3')
**oGB343**	ACAGCTATGACATGATTACGCTGTGGTGATCCGTGCG
**oCJ746**	CTACGACGCTCCGTTGTCCT
**oCJ747**	CTTACCCACCGCTAGGCCC
**oCJ748**	ACGACGGCCAGTGCCAAGATGCGCCAAACCTTGTAGG
**oLC0059**	CGTATGTGCATGAGCGTTAACTTACCCACCGCTAGGCCCCG
**oLC0060**	TCCGAATTCTTATTCGACATCCCGGGACCTCCTTCGGTGT

**oLC0099**	GTAAGACCGGTTTCGACAGT
**oLC0100**	CTGCGGCAGCCAAGAAACCT
**CJ_phaC1437**	AACGGAGCGTCGTAGGAGCTGTTGACAATTAATCATCGGCTCGTATAATGTGTGGAATTGTGAGCG<br>GATAACAATTTCACACCGAAGGAGGTCCCGGGATGAGTAACAAGAGTAACGATGAGTTGAAGTATC<br>AAGCCTCTGAAAACACCTTGGGGCTTAATCCTGTCGTTGGGCTGCGTGGAAAGGATCTACTGGCTT<br>CTGCTCGAATGGTGCTTAGGCAGGCCATCAAGCAACCGGTGCACAGCGTCAAACATGTCGCGCACT<br>TTGGTCTTGAACTCAAGAACGTACTGCTGGGTAAATCCGGGCTGCAACCGACCAGCGATGACCGTC<br>GCTTCGCCGATCCGGCCTGGAGCCAGAACCCGCTCTATAAACGTTATTTGCAAACCTACCTGGCGT<br>GGCGCAAGGAACTCCACGACTGGATCGATGAAAGTAACCTCGCCCCCAAGGATGTGGCGCGTGGGC<br>ACTTCGTGATCAACCTCATGACCGATGCCATGGCGCCGACCAACACCGCGGCCAACCCGGCGGCAG<br>TCAAACGCTTTTTCGAAACCGGTGGCAAAAGCCTGCTCGACGGCCTCTCGCACCTGGCCAAGGATC<br>TGGTACACAACGGCGGCATGCCGAGCCAGGTCAACATGGGTGCATTCGAGGTCGGCAAGAGCCTGG<br>GCGTGACCGAAGGCGCGGTGGTGTTTCGCAACGATGTGCTGGAACTGATCCAGTACAAGCCGACCA<br>CCGAGCAGGTATACGAACGCCCGCTGCTGGTGGTGCCGCCGCAGATCAACAAGTTCTACGTTTTCG<br>ACCTGAGCCCGGACAAGAGCCTGGCGCGGTTCTGCCTGCGCAACAACGTGCAAACGTTCATCGTCA<br>GCTGGCGAAATCCCACCAAGGAACAGCGAGAGTGGGGCCTGTCGACCTACATCGAAGCCCTCAAGG<br>AAGCGGTTGACGTCGTTACCGCGATCACCGGCAGCAAAGACGTGAACATGCTCGGGGCCTGCTCCG<br>GCGGCATCACTTGCACTGCGCTGCTGGGCCATTACGCGGCGATTGGCGAAAACAAGGTCAACGCCC<br>TGACCTTGCTGGTGACCGTGCTTGATACCACCCTCGACAGCGACGTCGCCCTGTTCGTCAATGAAC<br>AGACCCTTGAAGCCGCCAAGCGCCACTCGTACCAGGCCGGCGTACTGGAAGGCCGCGACATGGCGA<br>AGGTCTTCGCCTGGATGCGCCCCAACGATCTGATCTGGAACTACTGGGTCAACAATTACCTGCTAG<br>GCAACGAACCGCCGGTGTTCGACATCCTGTTCTGGAACAACGACACCACACGGTTGCCCGCGGCGT<br>TCCACGGCGACCTGGTCGAACTGTTCAAAAATAACCCACTGATTCGCCCGAATGCACTGGAAGTGT<br>GCGGCACCCCCATCGACCTCAAGCAGGTGACGGCCGACATCTTTTCCCTGGCCGGCACCAACGACC<br>ACATCACCCCGTGGAAGTCCTGCTACAAGTCGGCGCAACTGTTTGGCGGCAACGTTGAATTCGTGC<br>TGTCGAGCGGCGGGCATATCAAGAGCATCCTGAACCCGCCGGGCAATCCGAAATCGCGCTACATGA<br>CCAGCACCGAAGTGGCGGAAAATGCCGATGAATGGCAAGCGAATGCCACCAAGCATACAGATTCCT<br>GGTGGCTGCACTGGCAGGCCTGGCAGGCCCAACGCTCGGGCGAGCTGAAAAAGTCCCCGACAAAAC<br>TGGGCAGCAAGGCGTATCCGGCAGGTGAAGCGGCGCCAGGCACGTACGTGCACGAACGGTAACTTA<br>CCCACCGCTAG

Table S4. Polymer properties of PHDU-6 used in this study, taken from Cywar *et al.*[3]

Property	Value
M_n (kg mol^{-1})	172
$Đ$	1.60
T_g (°C)	-46
T_m (°C)	34, 60
$T_{d,5\%}$ (°C)	288
Young's modulus (MPa)	4.58 ± 0.21 MPa
Tensile stress (MPa)	14.2 ± 1 MPa
Tensile strain (%)	379 ± 38%
E'_{max} (-75 °C, MPa)	2063 ± 91
E'_{RT} (23 °C, MPa)	166 ± 16

Table S5. Average tensile stress/strain values for each reported sample.

Polymer	Tensile strength (σ_B, MPa)	Tensile strain (ε_B, %)	Young's modulus (*E*, MPa)
PHBU-5	20.30 ± 1.50	6.6 ± 0.8	833 ± 121
$PHBU_3$-*blend*-$PHDU_1$	15.10 ± 0.66	21.9 ± 4.4	665 ± 53
$PHBU_1$-*blend*-$PHDU_1$	6.63 ± 0.14	93.9 ± 13.6	70 ± 13
$PHBU_1$-*blend*-$PHDU_3$	1.97 ± 0.31	187.0 ± 33.0	6 ± 1
$PHBU_3$-*blend*-$PHDU_1$ Rx1	15.80 ± 1.48	25.8 ± 5.0	647 ± 82
$PHBU_1$-*blend*-$PHDU_1$ Rx1	6.89 ± 0.17	109.0 ± 15.0	50 ± 9
$PHBU_1$-*blend*-$PHDU_3$ Rx1	1.19 ± 0.20	266.0 ± 30.0	1.5 ± 0.2

Table S6. Tabulated DSC data of PHBU-5 and its blends

Polymer	T_g (°C)	T_m (°C)	T_{cc} (°C)
PHBU-5	-5.1	150	60
$PHBU_3$-*blend*-$PHDU_1$	-39, -0.1	158	37
$PHBU_1$-*blend*-$PHDU_1$	-39, -3.9	156	75
$PHBU_1$-*blend*-$PHDU_3$	-39, -3.9	-	-

Table S7. Tabulated DMA storage modulus (*E*') values for PHBU and each blend at low temperature (E'_{max}, −75 ˚C) and room temperature (E'_{RT}, 23 ˚C).

Polymer	E'_{maz} (−75 °C, MPa)	E'_{RT} (23 °C, MPa)
PHBU-5	8134	2271
$PHBU_3$-*blend*-$PHDU_1$	4887	1268
$PHBU_1$-*blend*-$PHDU_1$	3135	93
$PHBU_1$-*blend*-$PHDU_3$	2941	12

Table S8. CHN Elemental Analysis results for samples used for biodegradation test.

Sample Name		Results		
		%N	%C	%H
PHBU-5	1	0	57.60	7.41
	2	0	57.24	7.42
	3	0	58.22	7.53
	Average	0	57.69	7.45
	Std	0	0.50	0.07
$PHBU_1$-*blend*-$PHDU_3$	1	0	64.37	9.14
	2	0	63.93	9.07

	3	0	64.93	9.22
	Average	0	64.41	9.14
	Std	0	0.50	0.08
$PHBU_3$-*blend*-$PHDU_1$	1	0	59.08	7.88
	2	0	59.21	7.89
	3	0	60.29	8.04
	Average	0	59.53	7.94
	Std	0	0.66	0.09

Table S9. Summary of estimated lifetime of PHBU film samples using first order kinetic model.

**Sample**	**Rate constant (day^{-1})**	**Estimated 90% biodegradation time (day)**	**R^2**
PHBU-5	0.001891	1182	0.9733
$PHBU_1$-*blend*-$PHDU_3$	0.001741	1295	0.9859
$PHBU_3$-*blend*-$PHDU_1$	0.002209	1022	0.9727

Supplementary figures

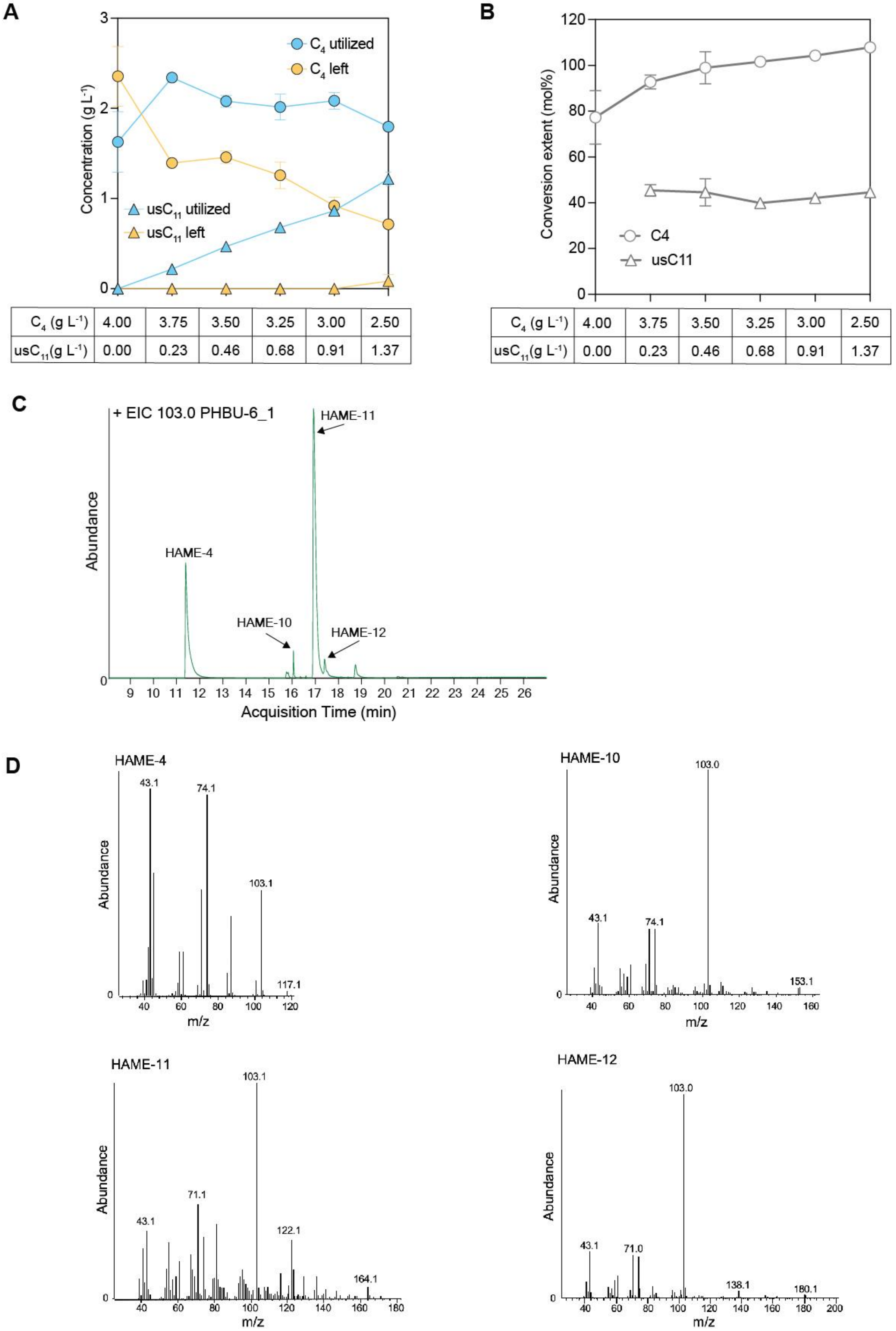

Figure S1. Shake flasks experiment in 500 mL flasks**. A)** Shake flasks profiles, C_4 represents sodium butyrate, usC_{11} represents 10-undecenoic acid. The concentrations of usC_{11} were calculated based on the density value of 0.912 g mL^{-1} at 25 °C, considering volume concentrations of 0, 0.25, 0.5, 0.75, 1.00, 1.50 mL L^{-1}. Error bars represent the standard deviation calculated from biological triplicates. **B)** Conversion extent of sodium butyrate (C_4) and 10-undecenoic acid (usC_{11}) to PHBU in the shake flasks experiments. Error bars represent the standard deviation calculated from biological triplicates. **C)** Extracted ion chromatograms (EIC) at mass-to-charge ratio (m/z) 103, of sample PHBU-6_1, corresponding to the 1st sample among the triplicates of 2.5 g/L sodium butyrate in Fig. S1A. Four peaks represent HAME-4, 10, 11, and 12, respectively. D) Mass spectrometry graphs illustrating the abundance of various m/z at each of the four retention time points corresponding to the signal peaks of HAME-4, 10, 11, and 12.

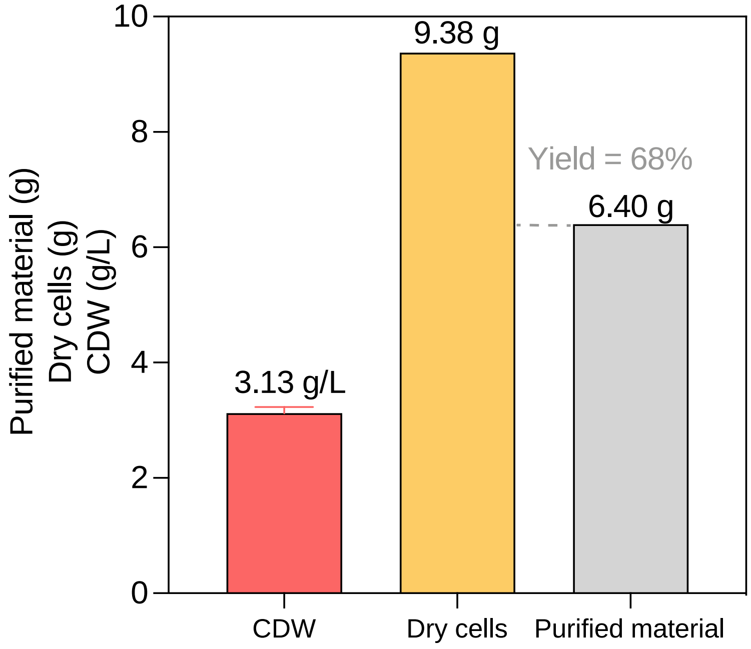

Figure S2. Profiles of shake flasks experiment in 2.8L flasks. Error bar in CDW represents standard deviation of 6 biological replicates. The yield was calculated as the mass of purified material divided by the mass of combined dry cells: 6.40/9.38*100.

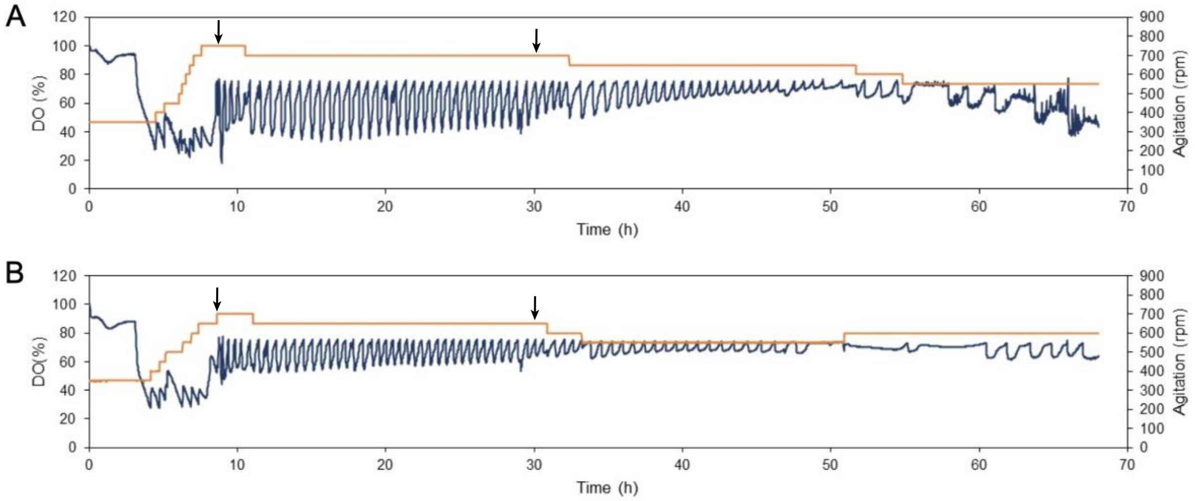

Figure S3. Dissolved oxygen (DO) and agitation profiles, panels A and B represent profiles from two independent bioreactors. The data was collected during PHBU production in modified minimal M9 medium and DO-stat fed-batch mode. Black arrows indicate the time at which sodium butyrate (2 g L^{-1}) and 10-undecenoic acid (0.16 g L^{-1}) were added in the bioreactors. These profiles correspond to the bioreactor results shown in Fig. 3D.

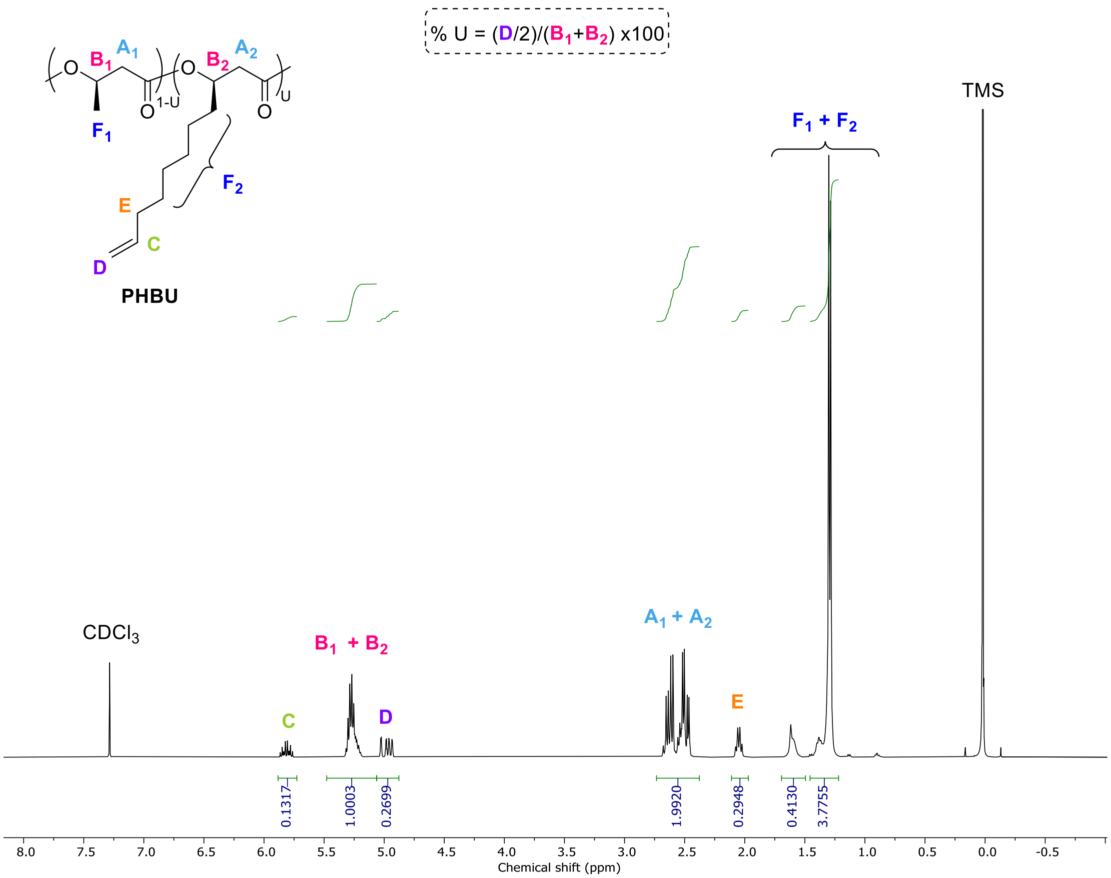

Figure S4. Example ^{1}H NMR spectrum ($CDCl_3$, 400 MHz) of PHBU and calculation of % U. %U was determined by comparing the integrals of the resonances of the alkene (**D**, 2H) with those of the polymer backbone (**B_1+B_2**, 1H), using the equation (**D**/2)/(**B_1+B_2**)×100.

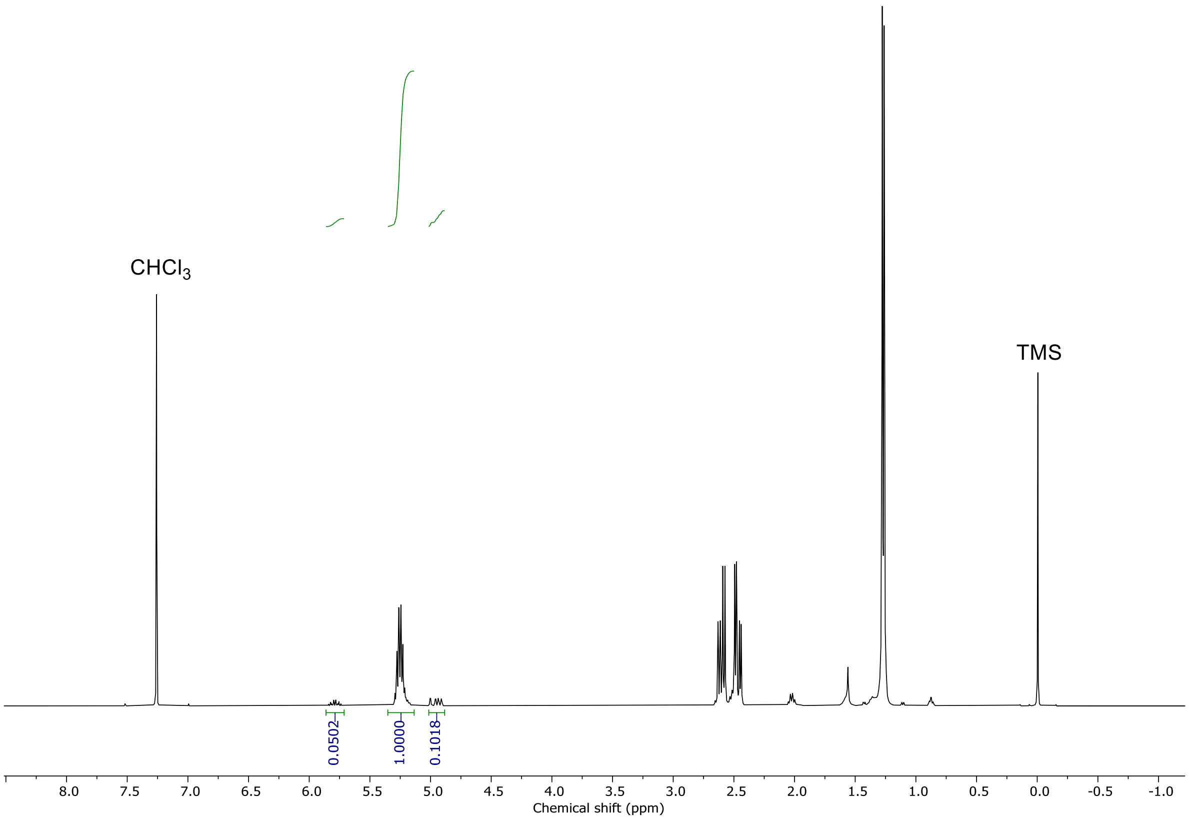

Figure S5. ^{1}H NMR ($CDCl_3$, 400 MHz) spectrum of PHBU-5. See Figure S4 for annotations.

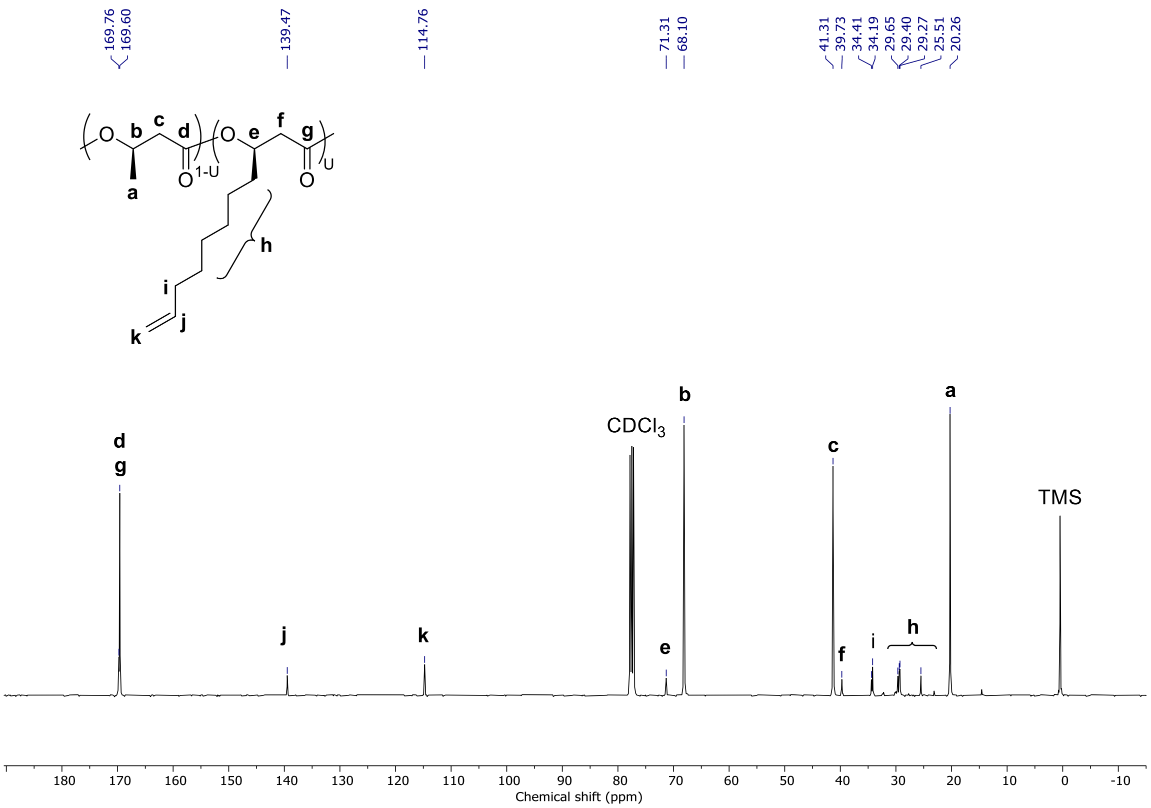

Figure S6. $^{13}C\{^{1}H\}$ NMR ($CDCl_3$, 101 MHz) spectrum of PHBU-5.

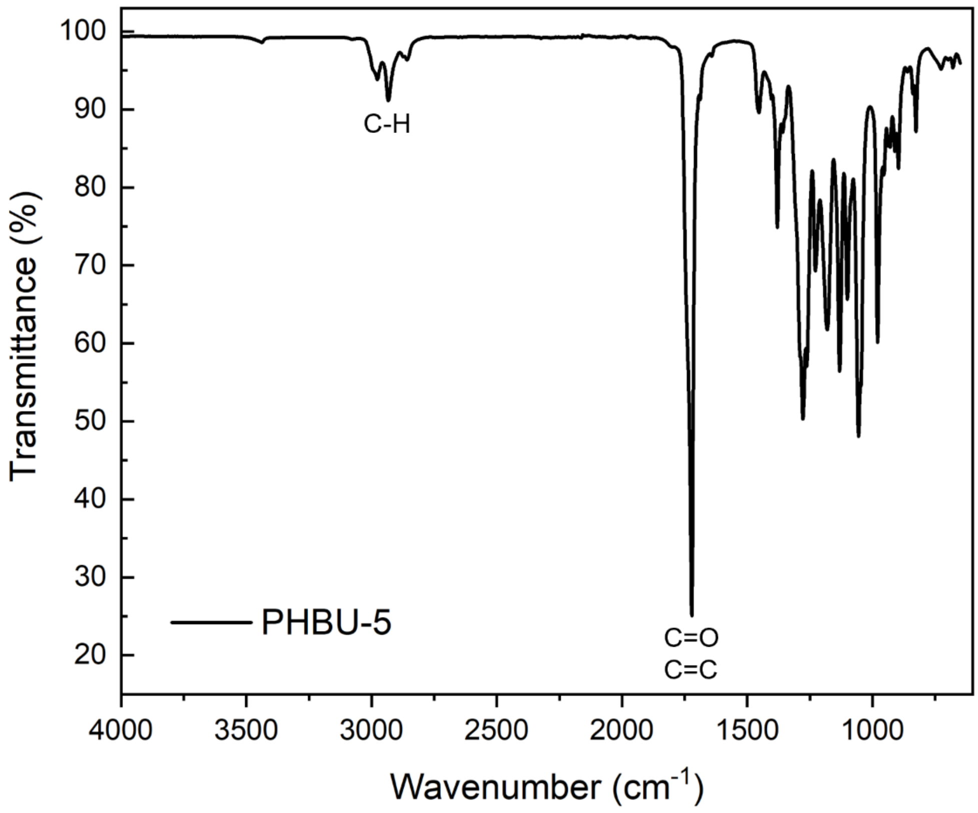

Figure S7. FTIR spectrum of PHBU-5.

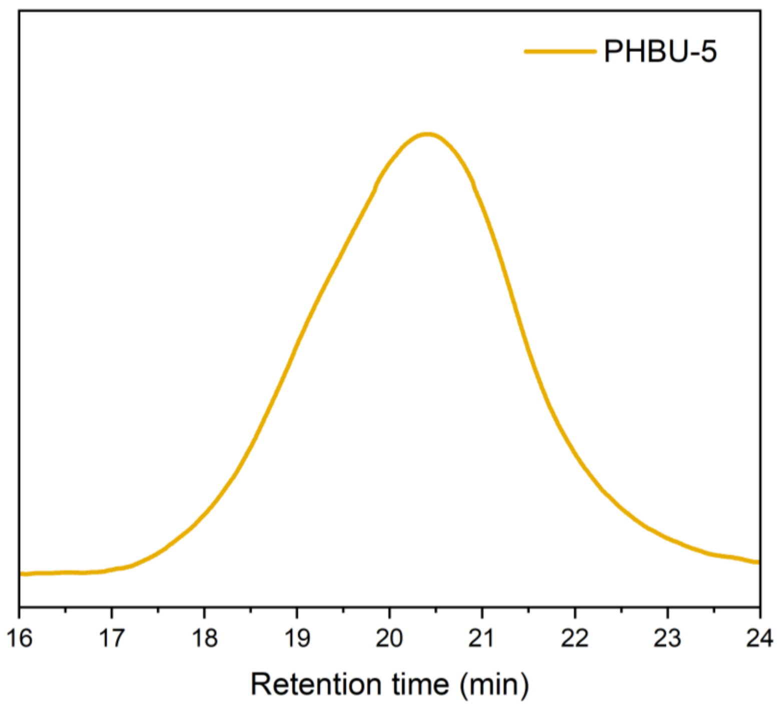

Figure S8. $CHCl_3$ GPC trace of PHBU-5.

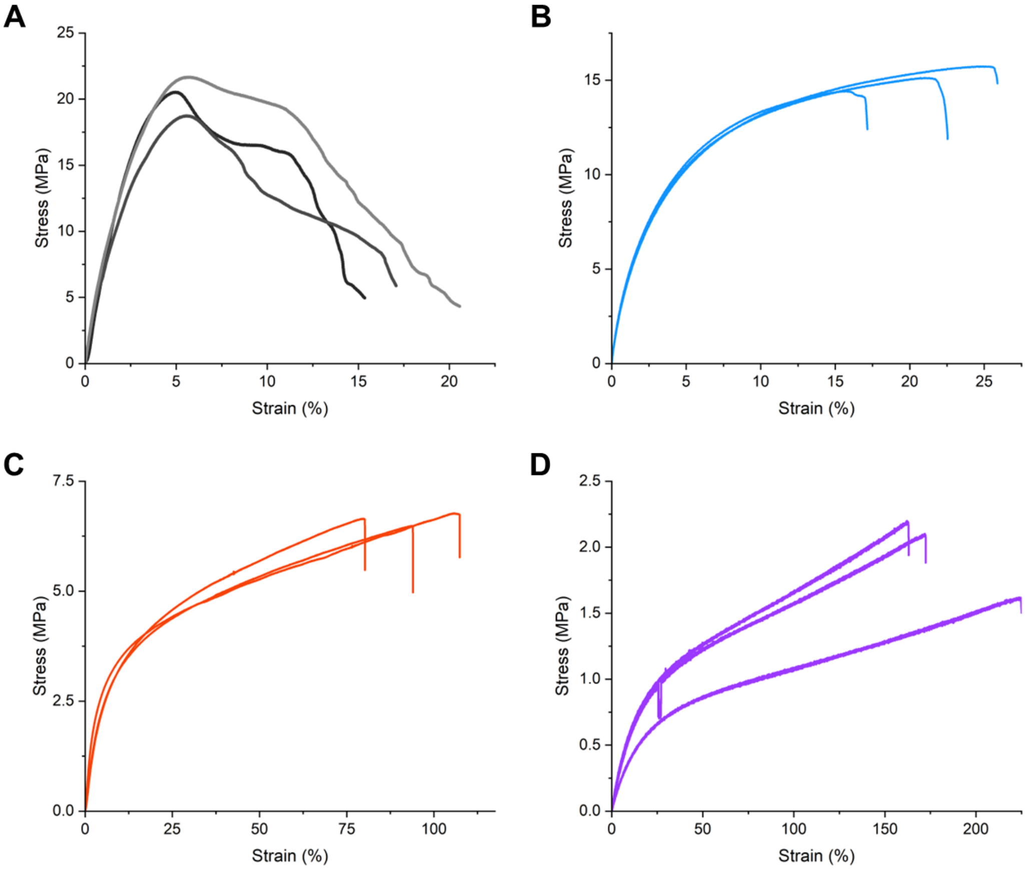

Figure S9. Individual stress/strain curves (~23 ˚C, 5 mm min^{-1}) for **A**) PHBU-5, **B**) $PHBU_3$-*blend*-$PHDU_1$ **C**) $PHBU_1$-*blend*-$PHDU_1$ and **D**) $PHBU_1$-*blend*-$PHDU_3$.

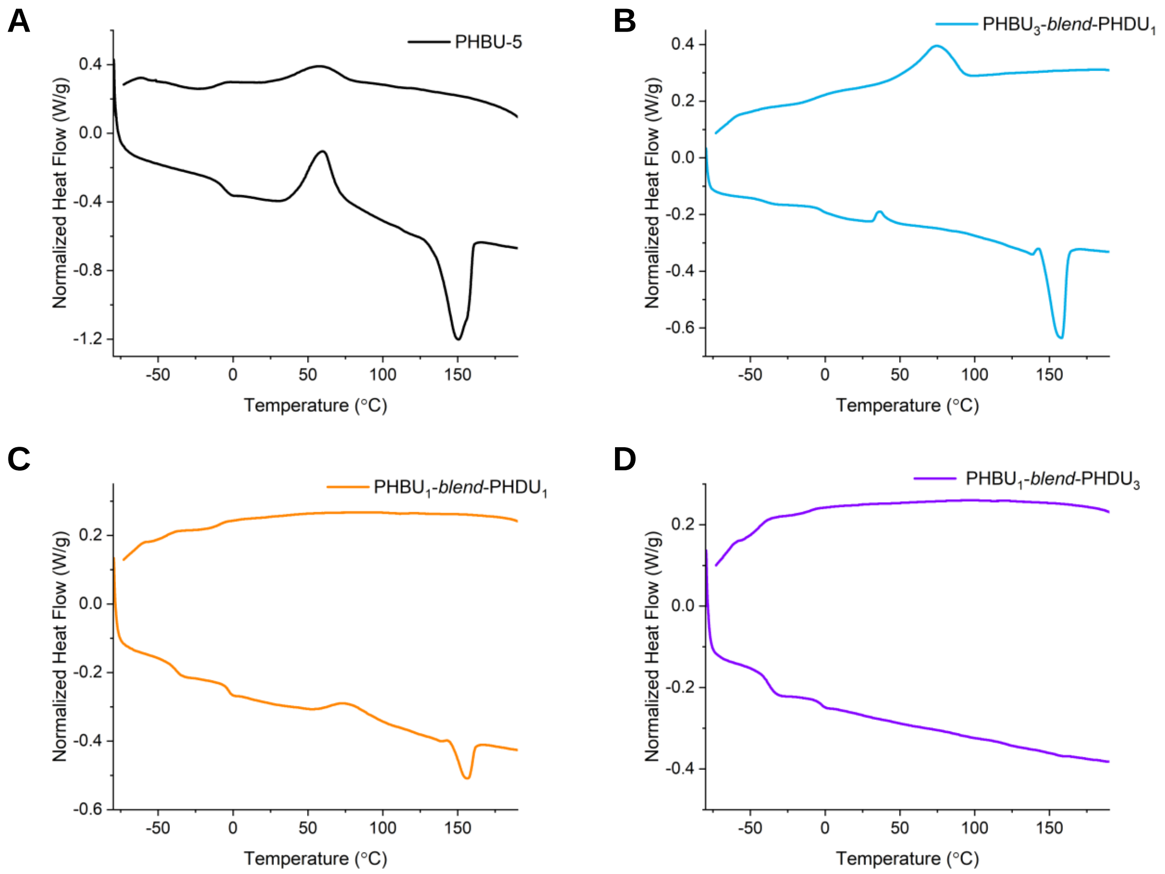

Figure S10. DSC traces (second heating scan) for **A**) PHBU-5, **B**) $PHBU_3$-*blend*-$PHDU_1$ **C**) $PHBU_1$-*blend*-$PHDU_1$ and **D**) $PHBU_1$-*blend*-$PHDU_3$.

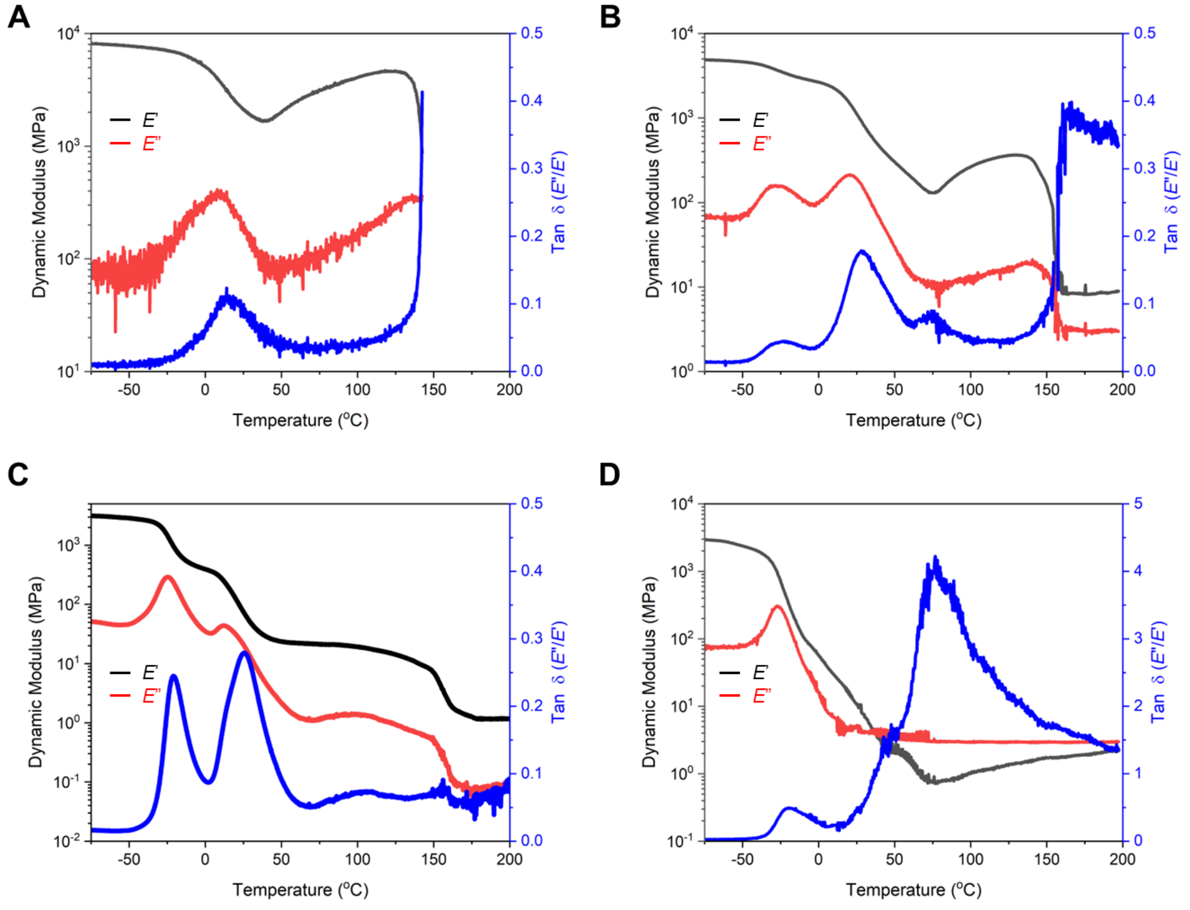

Figure S11. Individual DMA thermograms (-75 ˚C to 200 ˚C or fail, 3 ˚C min^{-1}, 15 µm, 1 Hz) for **A**) PHBU-5, **B**) $PHBU_3$-*blend*-$PHDU_1$ **C**) $PHBU_1$-*blend*-$PHDU_1$ and **D**) $PHBU_1$-*blend*-$PHDU_3$.

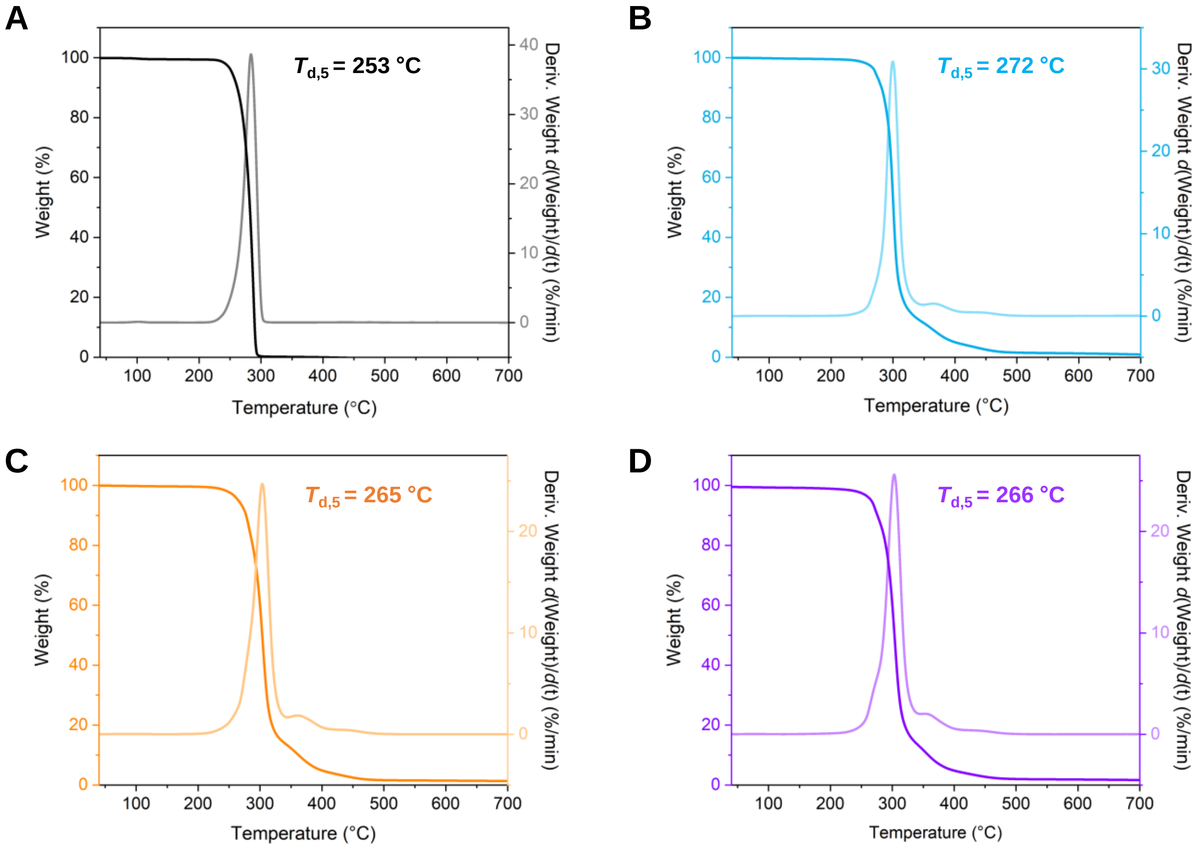

Figure S12. TGA graphs for **A**) PHBU-5, **B**) $PHBU_3$-*blend*-$PHDU_1$ **C**) $PHBU_1$-*blend*-$PHDU_1$ and **D**) $PHBU_1$-*blend*-$PHDU_3$.

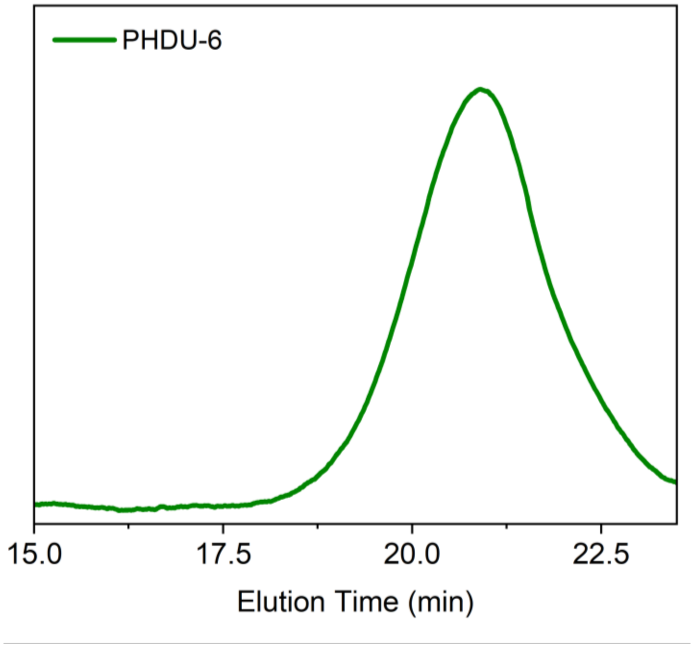

Figure S13. $CHCl_3$ GPC trace of PHDU-6.

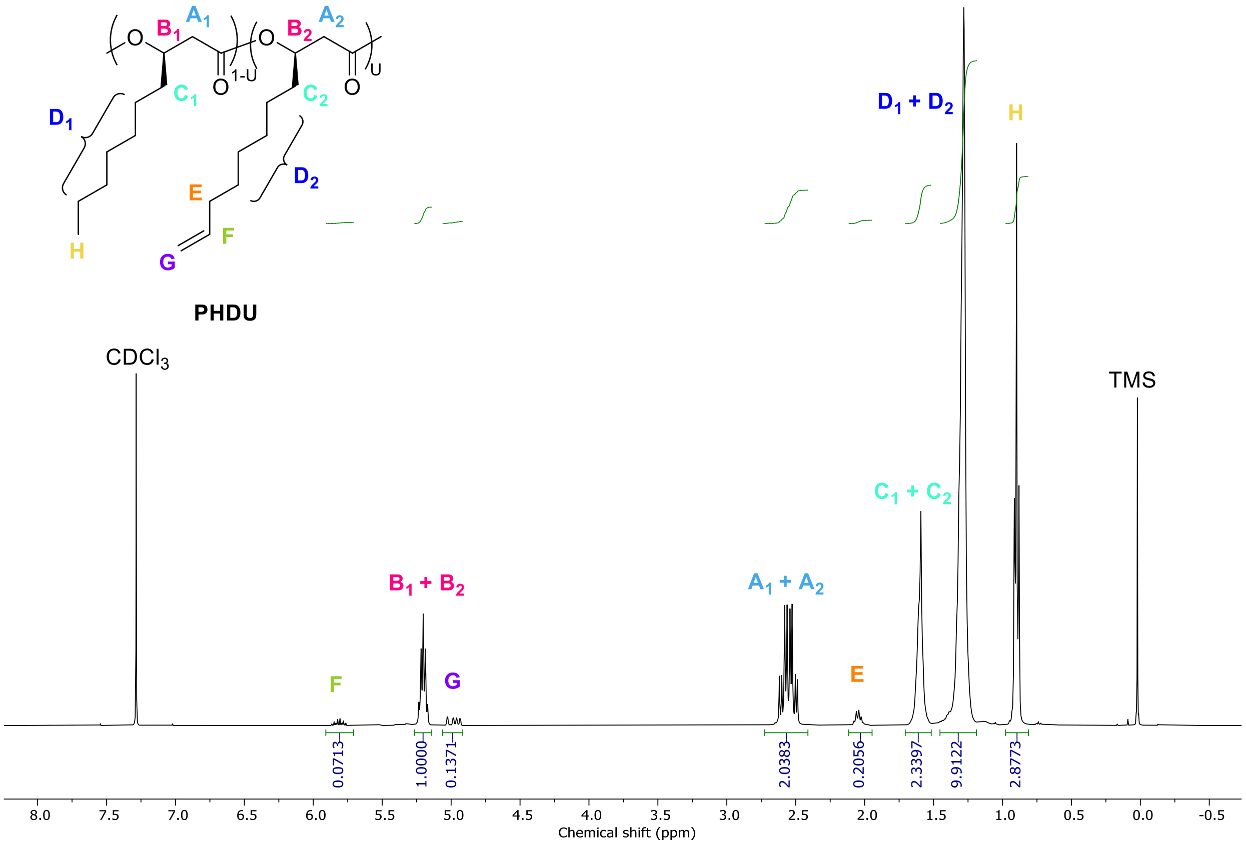

Figure S14. ^{1}H NMR spectrum ($CDCl_3$, 400 MHz) of PHDU-6.

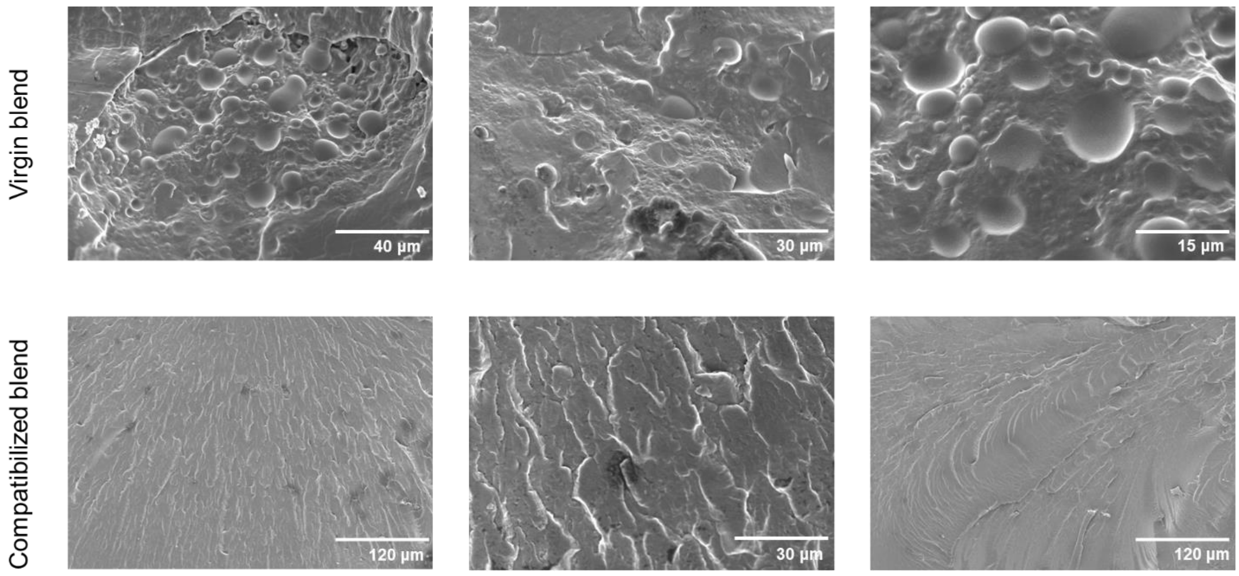

Figure S15. Additional SEM images of film cross-sections for virgin (top) and compatibilized (bottom) $PHBU_1$-*blend*-$PHDU_3$.

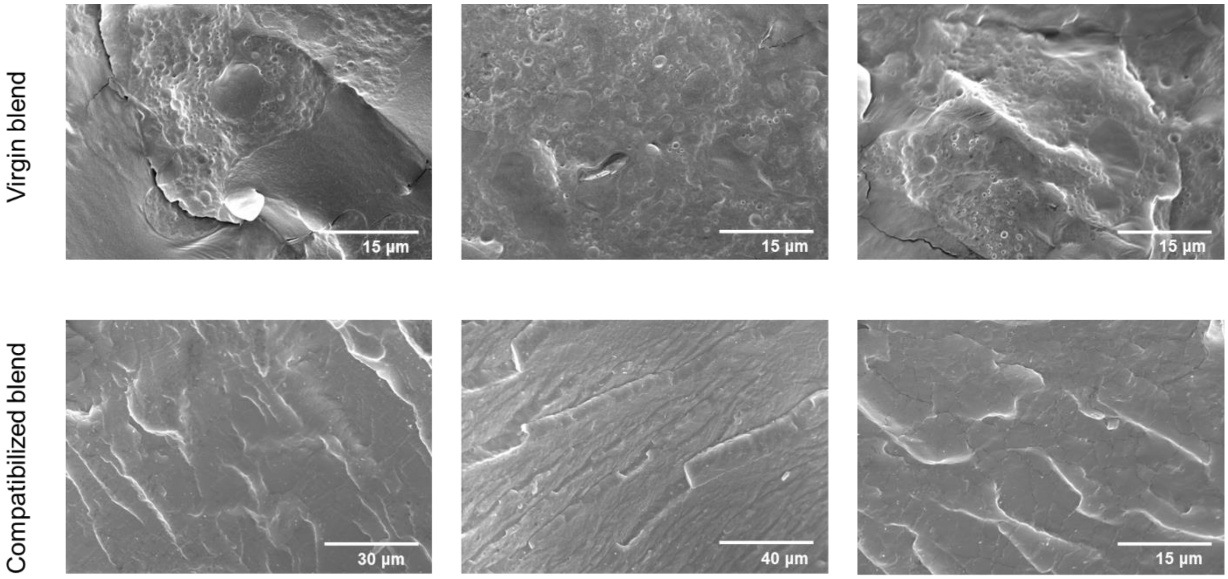

Figure S16. Additional SEM images of film cross-sections for virgin (top) and compatibilized (bottom) $PHBU_1$-*blend*-$PHDU_1$.

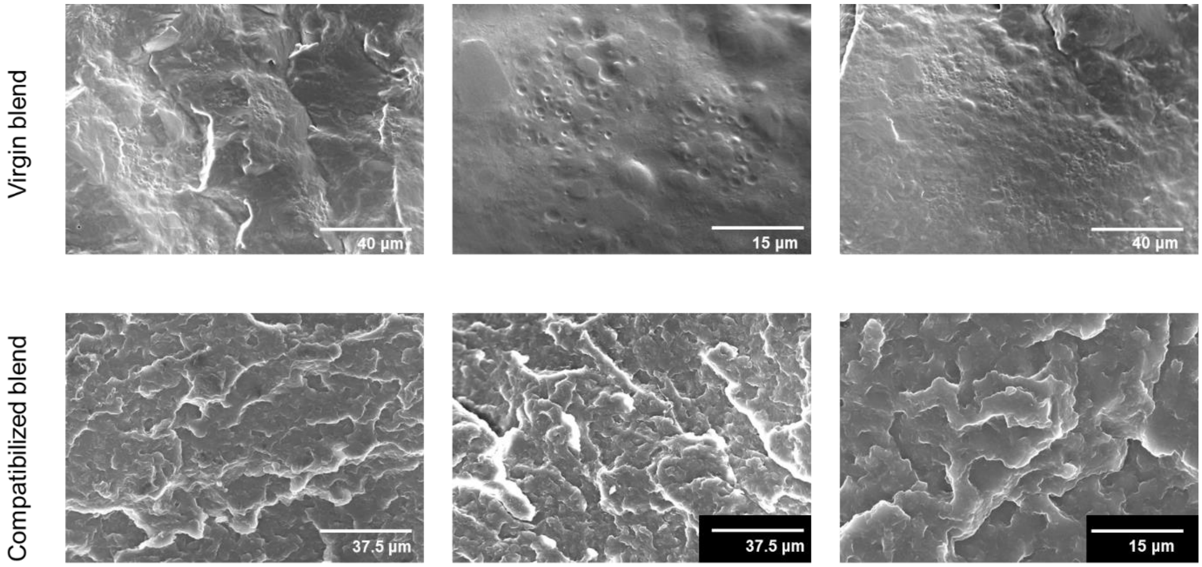

Figure S17. Additional SEM images of film cross-sections for virgin (top) and compatibilized (bottom) $PHBU_3$-*blend*-$PHDU_1$.

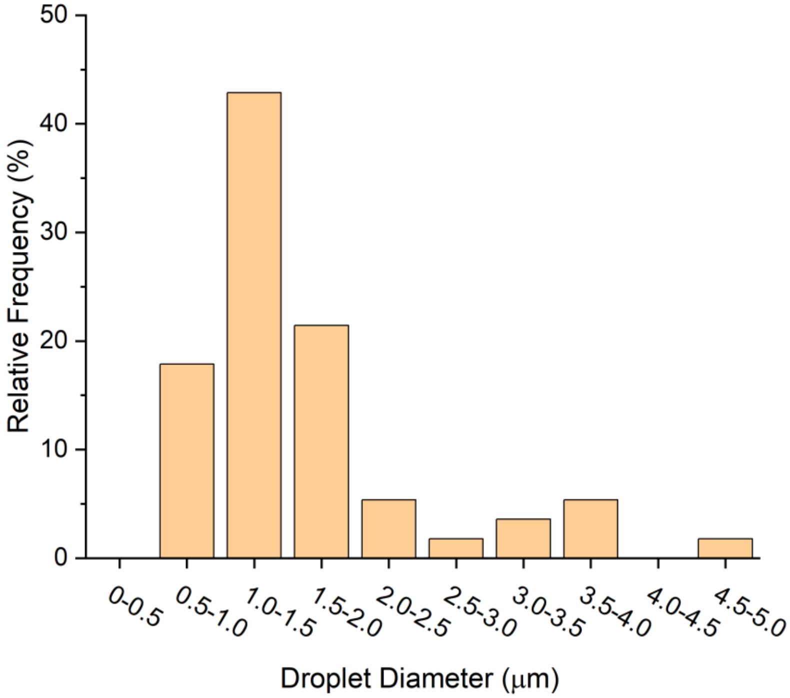

Figure S18. Droplet diameter analysis for PHBU$_1$-*blend*-PHDU$_3$ cross-sectional images.

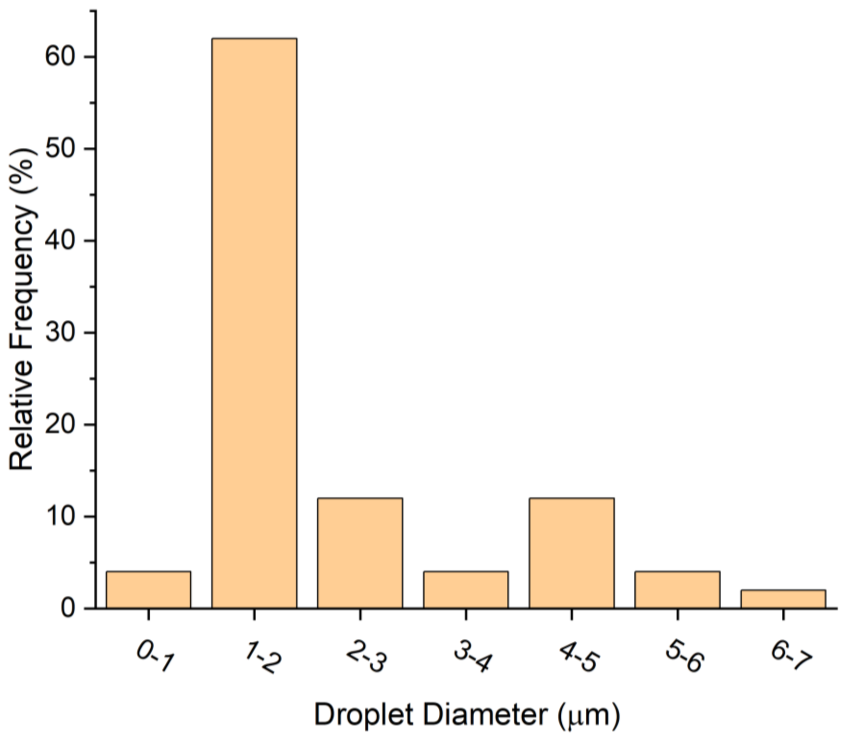

Figure S19. Droplet diameter analysis for PHBU$_1$-*blend*-PHDU$_1$ cross-sectional images.

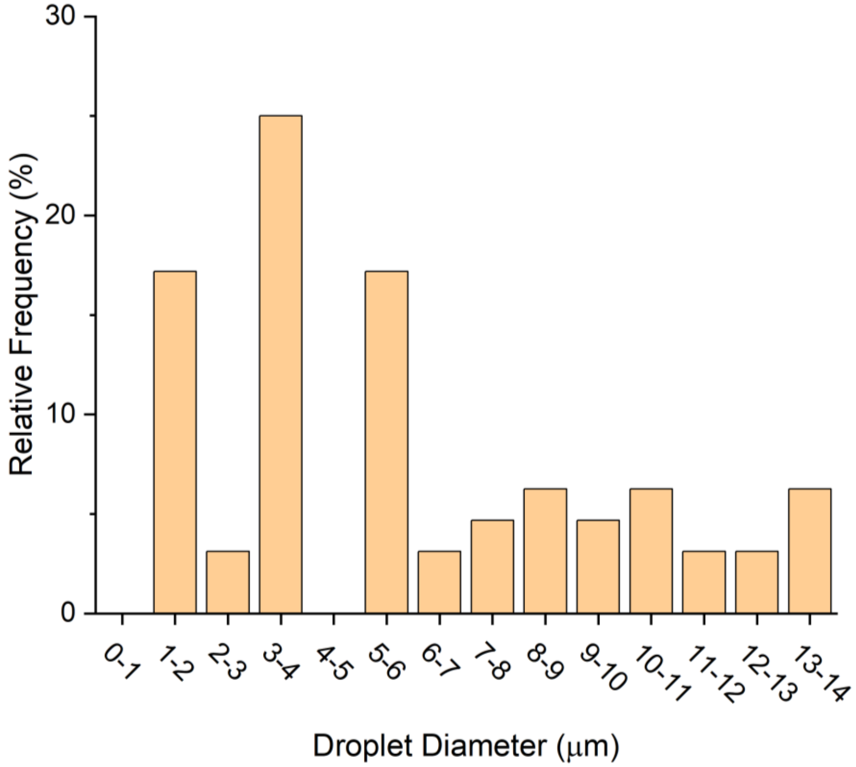

Figure S20. Droplet diameter analysis for PHBU$_3$-*blend*-PHDU$_1$ cross-sectional images.

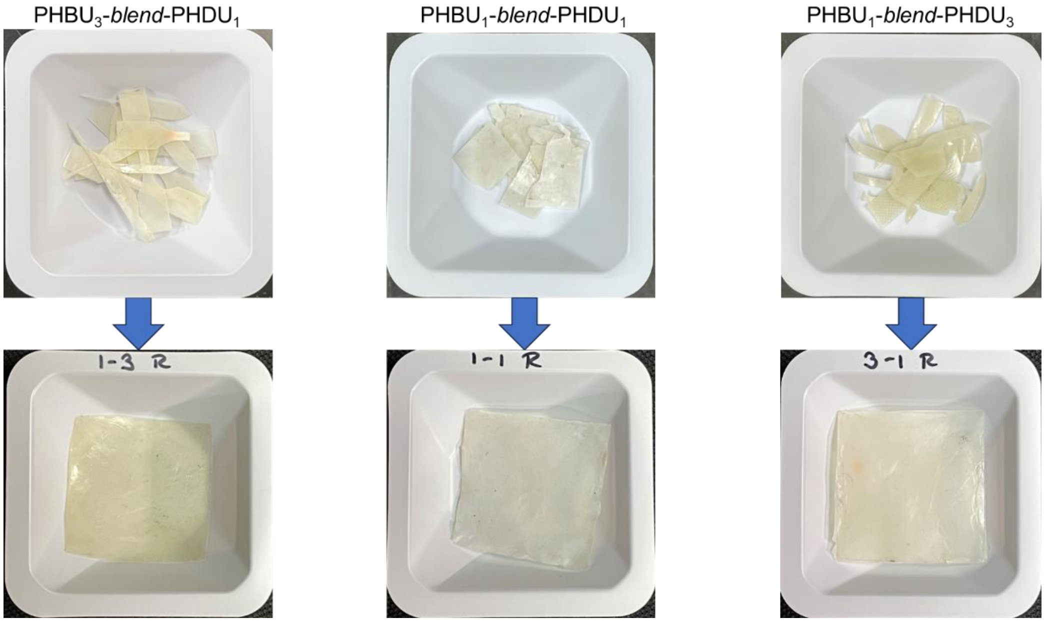

Figure S21. Digital images for (top) virgin PHBU$_3$-*blend*-PHDU$_1$ (left), PHBU$_3$-*blend*-PHDU$_1$ (middle), and PHBU$_3$-*blend*-PHDU$_1$ (right) tensile scraps, and (bottom) corresponding reprocessed films (165 ˚C, 5 min).

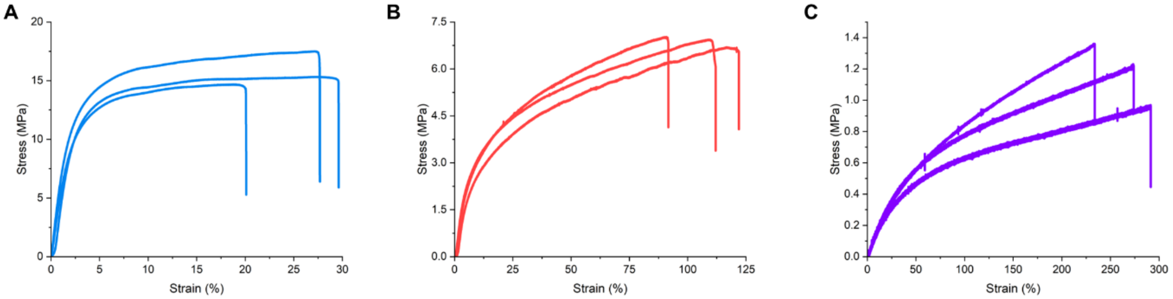

Figure S22. Individual tensile stress/strain (~23 ˚C, 5 mm min^{-1}) curves for reprocessed **A**) PHBU$_3$-*blend*-PHDU$_1$, **B**) PHBU$_1$-*blend*-PHDU$_1$, and **C**) PHBU$_1$-*blend*-PHDU$_3$.

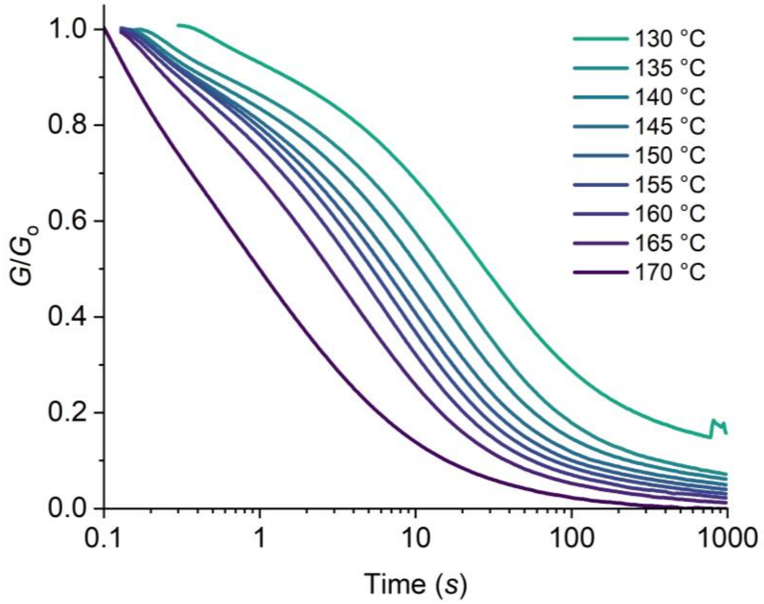

Figure S23. Normalized stress relaxation traces for $PHBU_3$-*blend*-$PHDU_1$ by shear rheology (130 °C to 170 °C, 5 °C increments, 5%, 1000 *s*, 0.1 *s* rise time).

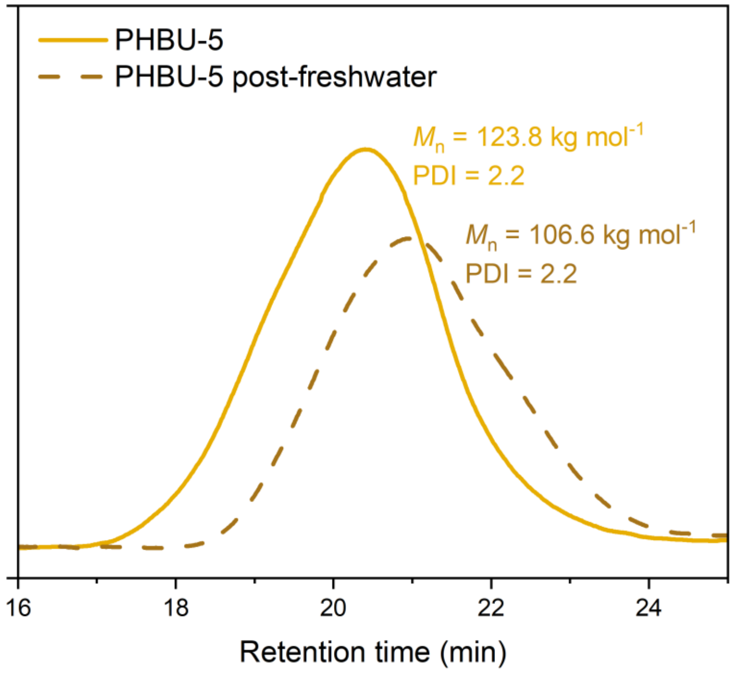

Figure S24. Overlay of GPC traces of PHBU-5 before and after freshwater degradation for 90 days.

References

1. Breuillac, A.; Kassalias, A.; Nicolaÿ, R. Polybutadiene Vitrimers Based on Dioxaborolane Chemistry and Dual Networks with Static and Dynamic Cross-links. *Macromolecules* **2019**, *52* (18), 7102-7113.
2. Johnson, C. W.; Beckham, G. T. Aromatic catabolic pathway selection for optimal production of pyruvate and lactate from lignin. *Metab Eng* **2015**, *28*, 240-247.
3. Cywar, R. M.; Ling, C.; Clarke, R. W.; Kim, D. H.; Kneucker, C. M.; Salvachúa, D.; Addison, B.; Hesse, S. A.; Takacs, C. J.; Xu, S.; et al. Elastomeric vitrimers from designer polyhydroxyalkanoates with recyclability and biodegradability. *Science Advances* **2023**, *9* (47), eadi1735.
4. Jayakody, L. N.; Johnson, C. W.; Whitham, J. M.; Giannone, R. J.; Black, B. A.; Cleveland, N. S.; Klingeman, D. M.; Michener, W. E.; Olstad, J. L.; Vardon, D. R.; et al. Thermochemical wastewater valorization via enhanced microbial toxicity tolerance. *Energy & Environmental Science* **2018**, *11* (6), 1625-1638, 10.1039/C8EE00460A.
